# Synthesis and Structure of an *o*‐Carboranyl‐Substituted Three‐Coordinate Borane Radical Anion

**DOI:** 10.1002/chem.202100938

**Published:** 2021-05-03

**Authors:** Johannes Krebs, Martin Haehnel, Ivo Krummenacher, Alexandra Friedrich, Holger Braunschweig, Maik Finze, Lei Ji, Todd B. Marder

**Affiliations:** ^1^ Institute for Inorganic Chemistry Institute for Sustainable Chemistry & Catalysis with Boron Julius-Maximilians-Universität Würzburg Am Hubland 97074 Würzburg Germany; ^2^ Frontiers Science Center for Flexible Electronics (FSCFE) Shaanxi Institute of Flexible Electronics (SIFE) Northwestern Polytechnical University 127 West Youyi Road 710072 Xi'an P. R. China

**Keywords:** carborane, charge transfer, radical, three-coordinate boron, Wade's rules

## Abstract

Bis(1‐(4‐tolyl)‐carboran‐2‐yl)‐(4‐tolyl)‐borane [(1‐(4‐MeC_6_H_4_)‐*closo*‐1,2‐C_2_B_10_H_10_‐2‐)_2_(4‐MeC_6_H_4_)B] (**1**), a new bis(*o*‐carboranyl)‐(R)‐borane was synthesised by lithiation of the *o*‐carboranyl precursor and subsequent salt metathesis reaction with (4‐tolyl)BBr_2_. Cyclic voltammetry experiments on **1** show multiple distinct reduction events with a one‐electron first reduction. In a selective reduction experiment the corresponding paramagnetic radical anion **1^.−^** was isolated and characterized. Single‐crystal structure analyses allow an in‐depth comparison of **1**, **1^.−^**, their calculated geometries, and the S_1_ excited state of **1**. Photophysical studies of **1** show a charge transfer (CT) emission with low quantum yield in solution but a strong increase in the solid state. TD‐DFT calculations were used to identify transition‐relevant orbitals.

## Graphical abstract

**Stabilization via three‐coordinate boron** allows the isolation of a paramagnetic radical anion of *ortho*‐carborane. A photophysical, electrochemical and crystallographic study backed by DFT calculations shines light on *closo*‐*nido* intermediate structures, charge transfer processes and electron accepting ability.

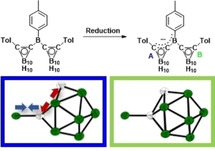

## Introduction

Three‐coordinate boron‐containing *π*‐conjugated molecules are of considerable interest. Boron, with its empty p_z_ orbital, is a strong acceptor, influencing the LUMO energy level, thereby tuning the HOMO‐LUMO gap.[^1^, ^2^, ^3^, ^4^, ^5^, ^6^, ^7^, ^8^, ^9^, ^10^, ^11^] Three‐coordinate boron has applications in linear[^8^, ^12^, ^13^, ^14^, ^15^, ^16^, ^17^, ^18^, ^19^, ^20^, ^21^, ^22^, ^23^, ^24^, ^25^, ^26^, ^27^, ^28^, ^29^, ^30^, ^31^, ^32^] and non‐linear optics,[^33^, ^34^, ^35^, ^36^, ^37^, ^38^, ^39^, ^40^, ^41^, ^42^, ^43^] bioimaging,[^45^, ^46^, ^47^, ^48^, ^49^, ^50^, ^51^, ^52^, ^53^, ^54^, ^55^, ^56^] sensors,[^57^, ^58^, ^59^, ^60^, ^61^] frustrated Lewis pairs (FLPs),[^62^, ^63^] and OLEDs.[^64^, ^65^] Dicarba‐*closo*‐dodecaboranes are a different class of boron‐based compounds that increasingly gain recognition in materials[^66^, ^67^, ^68^, ^69^, ^70^, ^71^] and pharmaceutical fields.[^72^, ^73^, ^74^, ^75^, ^76^] They are unique building blocks in optoelectronic materials with high thermal stability due to their 3‐dimensional aromaticity.[^77^, ^78^, ^79^, ^80^, ^81^, ^82^, ^83^] The *ortho*‐isomer is a rotation‐dependent *π*‐acceptor, accepting electron density into its C1−C2 σ*‐anti‐bonding orbital, often leading to aggregation‐induced emission (AIE).[^84^, ^85^, ^86^, ^87^, ^88^, ^89^, ^90^, ^91^, ^92^, ^93^, ^94^, ^95^, ^96^, ^97^, ^98^] Both aspects provide opportunities for novel material design. Close‐range conjugation of a carboranyl moiety to a 3‐coordinate boron has rarely been studied. Examples include *o*‐carboranyl‐substituted boron dihalides or acids for use in materials[^79^, ^99^, ^100^, ^101^] and more recent, crystallographic studies of C‐isopropyl‐*o*‐carborane‐organoboron derivatives with solid state geometries similar to 3‐coordinate boranes.^[102]^ *o*‐Carboranes substituted with C‐benzodiazaborolyl donor moieties were examined by photophysical, electrochemical, and spectroelectrochemical methods showing large Stokes shifts due to a substituent‐to‐cage charge transfer (CT) transition.[^8^, ^103^, ^104^, ^105^] Analogues of triarylboranes were synthesised using the BMes_2_ moiety as substituent and their spectro‐ and electrochemical properties were studied.^[106]^ Recently, 9‐borafluorene analogues of carborane‐fused “boroles” resulted in strong Lewis acidity while eliminating the rotational freedom of *ortho*‐carborane.[^107^, ^108^] A variety of B−N type borylated carboranes were reported featuring a strong interaction between boron and nitrogen.[^109^, ^110^, ^111^, ^112^, ^113^, ^114^] The elasticity of the C1−C2 bond of *o*‐carborane (1.629(6) Å) was studied in the presence of bulky substituents[^115^, ^116^, ^117^] (1.712(7) to 2.156(4) Å) and as a function of the C1−C2 σ*‐anti‐bonding orbital population by donating groups at C1 and C2 (1.723(2) to 2.065(7) Å).[^118^, ^119^, ^120^, ^121^, ^122^, ^123^, ^124^, ^125^, ^126^, ^127^] The interest in electrochemical reduction[^105^, ^128^, ^129^, ^130^, ^131^, ^132^] to populate this anti‐bonding orbital has led to its application in reversible electrochemical uranium capture.^[133]^ According to Wade's rules, such an increase of skeletal electrons (SE) results in a transition from a 2n+2 *closo*‐ towards a 2n+4 *nido*‐structure.[^134^, ^135^] Reports of rare 2n+3 SE structures have always sparked curiosity throughout the cluster community.[^136^, ^137^, ^138^, ^139^, ^140^, ^141^, ^142^, ^143^, ^144^, ^145^, ^146^, ^147^, ^148^] The 2n+3 *closo*‐*nido* cluster intermediates of *o*‐carboranes offer a structural comparison with calculated S_1_ CT excited state geometries as shown by Weber and Fox *et al*.[^104^, ^147^, ^149^] Reduction of *o*‐carboranes results in a 2n+3 SE system and typically requires a conjugated *π*‐system to stabilize the resulting radical.[^150^, ^151^, ^152^, ^153^] Few such crystal structures have been reported, all of which are dianionic[^144^, ^147^] or double bonded,^[146]^ resulting in diamagnetism. However, Weber and Fox *et al*. showed by a natural population analysis that the phenylene bridge in [1,4‐(1‐Ph‐*closo*‐1,2‐C_2_B_10_H_10_‐2‐)_2_‐C_6_H_4_]^2−^ is virtually neutral and, as such, the clusters can be considered to be two independent radicals.^[147]^ To the best of our knowledge, no crystallographic characterization of a paramagnetic *o*‐carboranyl monoanion has been reported. Herein, we report the synthesis and characterization of a new bis(*o*‐carboranyl)borane. The stabilizing effect of a 3‐coordinate borane enabled the isolation and crystallographic study of its paramagnetic monoanion. Similarities between the geometry of the 2n+3 SE *o*‐carboranyl radical anion and the optimised geometry of the S_1_ state of the neutral system help to understand the charge transfer mechanism and validate calculations thereof. Additionally, the combination of 3‐coordinate boron and *o*‐carborane in a single molecule reveals the relative acceptor strengths.

## Results and Discussion

### Synthesis, stability, and characterization

**Scheme 1:**
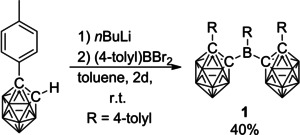
Synthesis of 3‐coordinate bis(1‐(4‐tolyl)‐carboran‐2‐yl)‐(4‐tolyl)‐borane (**1**).

Bis(1‐(4‐tolyl)‐carboran‐2‐yl)‐(4‐tolyl)‐borane (**1**) was chosen since the carboranyl moieties with their low steric demand enable free rotation at the C_cluster_−B_borane_ bonds and the 2‐fold substitution offers an intramolecular comparison on reduction or excitation. Lithiation of 1‐(4‐tolyl)‐*o*‐carborane^[110]^ and subsequent salt metathesis with dibromo(*p*‐tolyl)borane in toluene gave **1** in 40 % yield (Scheme 1). The yield of the reaction increased by a factor of 2 at room temperature for 2 days for step 2, compared to an overnight reaction at 80 °C. Compound **1** is unstable towards nucleophiles and decomposes in a solution in air within seconds. In the solid state, especially single crystals of a few hundred μm, decomposition takes up to 3 days under ambient conditions. Decomposition in the presence of moisture leads to 1‐(4‐tolyl)‐1,2‐dicarba‐*closo*‐dodecaborane and 4‐tolylboronic acid according to NMR spectroscopy and GC‐MS. Compound **1** was characterized by elemental analysis, HRMS, and NMR spectroscopy. The 3‐coordinate ^11^B NMR signal of **1** at 71.5 ppm is weak and broad (see Supporting Information). The downfield shift of the ^11^B NMR signals of the B_cluster_ atoms of **1** compared to the precursor were used to monitor reaction progress.

### Electrochemistry

**Figure 1:**
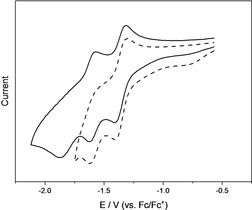
Cyclic voltammograms of **1** in CH_2_Cl_2_/0.1 M [*n*Bu_4_N][PF_6_] with a scan rate of 250 mVs^−1^. Both scans were normalized.

Redox potentials of **1** were measured by cyclic voltammetry in CH_2_Cl_2_ (Figure 1) showing a partially reversible one‐electron reduction at *E*_1/2_=−1.35 V (vs. Fc/Fc^+^), very similar to that of the related 1‐Ph‐2‐(Mes)_2_B‐*closo*‐1,2‐C_2_B_10_H_10_ (*E*_1/2_ in the range of −1.31 to −1.39 V).^[106]^ The second reduction at *E*_pc_=−1.63 V is clearly irreversible as shown by the dashed‐line scan in Figure 1, as is the third reduction at *E*_pc_=−1.87 V, which shows a corresponding oxidation peak at *E*_pa_=−1.57 V.

### Chemical reduction

**Scheme 2:**
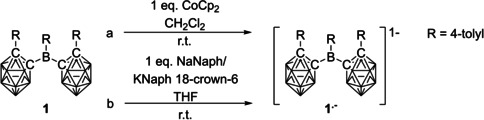
Reduction of **1**.

Several reduction protocols were employed to isolate the reduced form of **1** and a controlled and thus selective first reduction was achieved with Cp_2_Co in CH_2_Cl_2_ (*E* ^0^=−1.33 V vs. Fc/Fc^+^). The reduction product **CoCp_2_** ^**+**^ **1^.−^** was precipitated from CH_2_Cl_2_ with hexane at −30 °C (Scheme 2a). Reductions were also carried out in THF with elemental sodium, sodium naphthalenide (NaNaph), or potassium naphthalenide crown ether (K(18‐crown‐6)Naph) (Scheme 2b). A colour change from pale yellow to deep blue was observed upon addition of up to 1.0 equiv. of each reducing agent, which is attributed to the monoanion **1^.−^** (Scheme 2). All attempts to isolate the higher reduction products were unsuccessful, which seems to be in line with the observed irreversibility of the respective electrochemical reduction events.

### EPR spectroscopy

**Figure 2:**
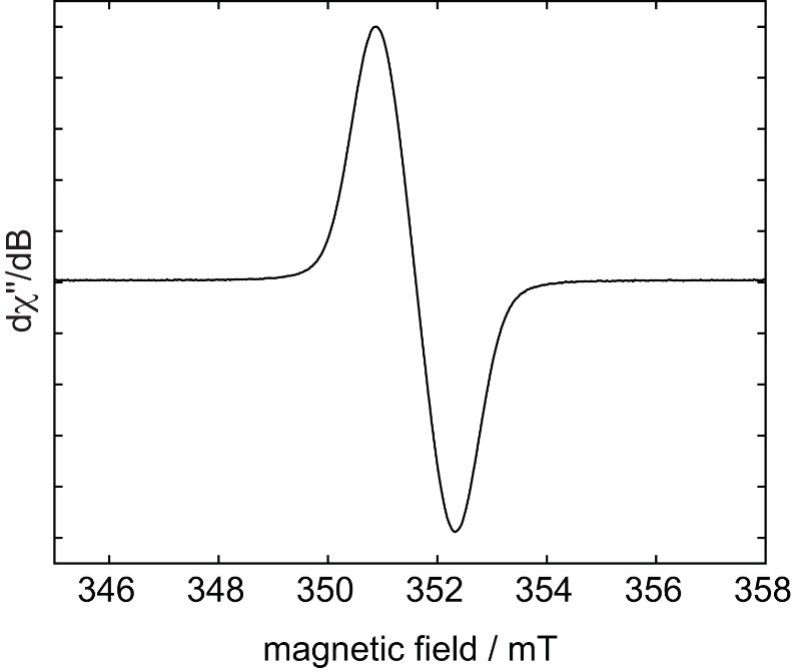
Experimental CW X‐band (9.85 GHz) EPR spectrum of **1^.−^** in THF at room temperature. Isotropic *g* value=2.0026 and peak‐to‐peak linewidth=1.4 mT.

The X‐band (9.85 GHz) EPR spectrum of **1^.−^** in THF (Figure 2) shows a broad single line of 1.4 mT width, centred close to the free electron g value g_i_ of 2.0023. The lack of resolved hyperfine couplings, even at low temperatures, points to significant delocalisation of the unpaired electron spin density over one or both clusters as has been observed and simulated for *o*‐carborane‐centred radicals.[^130^, ^154^]

### Single‐crystal structure analysis

**Figure 3:**
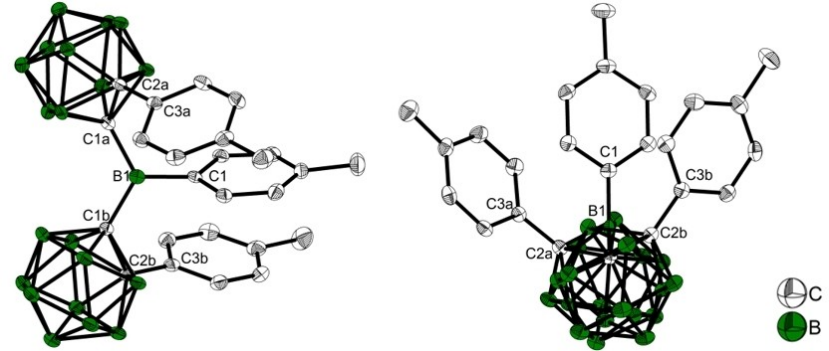
Molecular structure of **1** in the solid state at 100 K. H atoms are omitted for clarity. Thermal ellipsoids are drawn at 50 % probability. Left, view perpendicular to the 3‐coordinate boron plane; right, view along C1b−B1−C1a.

**Figure 4:**
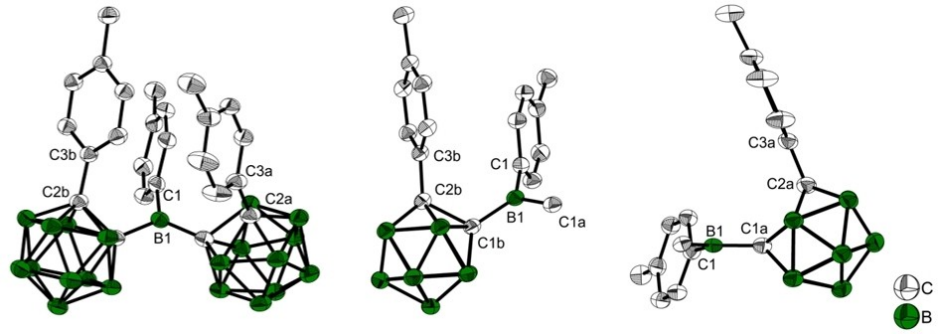
Molecular structure of **{K[18]crown‐6 ⋅ (THF)_2_}^+^1^.−^** in the solid state at 100 K. H atoms, solvent molecules, and cations are omitted for clarity. Thermal ellipsoids are drawn at 50 % probability. Left, view of the full molecule; middle, view perpendicular to the C1b−C2b bond with (*p*‐tolyl)carborane **a** omitted for clarity; right, view perpendicular to the C1a−C2a bond with (*p*‐tolyl)carborane **b** omitted for clarity.

**Figure 5:**
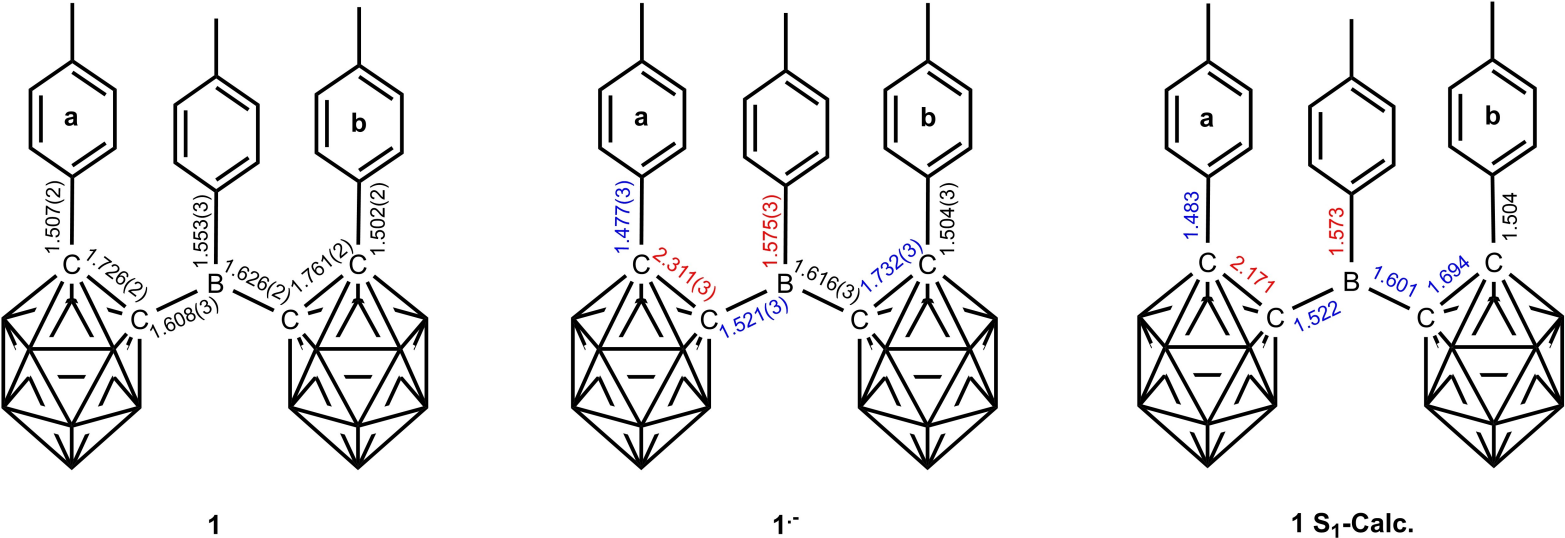
Bond lengths in **1**, **1^.−^** and the optimised S_1_ state of **1**. Red: increase in bond length, blue: decrease in bond length.

Single crystals of **1** suitable for X‐ray diffraction were grown from a saturated toluene solution at −30 °C, and crystals of **Na^+^1^.−^**, **CoCp_2_** ^**+**^ **1^.−^**, and **{K[18]crown‐6⋅(THF)_2_}^+^1^.−^** were grown by hexane diffusion into saturated THF solutions at −30 °C. The crystal structure of **{K[18]crown‐6⋅(THF)_2_}^+^1^.−^** is of high quality, while those of **Na^+^1^.−^** and **CoCp_2_** ^**+**^ **1^.−^** are of poorer quality and thus, are presented as confirmation of the monoanions in the Supporting Information, only. For consistency, the *o*‐carboranyl moiety with the C2_cluster_−C1_cluster_−B1−C1_tolyl_ dihedral angle closest to 90° with respect to the 3‐coordinate borane plane is labelled **a**, which is important for comparisons due to the interaction between the p_z_‐orbital of B1 and the C1−C2 σ*‐anti‐bonding orbital of *o*‐carborane. A comparison of the neutral and reduced structures and their respective calculated geometries and the calculated geometry of the S_1_ state of **1** are listed in Table 1 and in the text as (experimental(esd)/calculated Å). The two *o*‐carboranyl moieties in **1** adopt a *transoid* conformation with dihedral angles C2a−C1a−B1−C1 (62.4(2)/65.1°) and C2b−C1b−B1−C1 (−44.0(2)/−43.3°) (Figure 3, right). The *o*‐carboranyl moiety **a** has a greater overlap of C1−C2 *σ*‐ and B1‐p_z_ orbitals. The 3‐coordinate boron atom has a nearly ideal trigonal planar configuration, with the sum of the C−B−C angles being 359.8(3)/359.9°. The B1−C1 bond length to the tolyl‐moiety (1.553(3)/1.551 Å) is as expected (avg B(3)−Ar bond=1.556 Å).^[155]^ The B1−C1b (1.626(2)/1.605 Å) bond is significantly longer than the B1−C1a (1.608(3)/1.610 Å) bond in the solid state, while this difference is not present in the computed bond lengths. The C1b−C2b (1.761(2)/1.758 Å) bond is longer by ca. 0.035 Å (calc. 0.050 Å) than the C1a−C2a (1.726(2) Å/1.708 Å) bond both in the solid state and the computed molecule. Bond lengthening is observed for bonds involving tolyl groups with the smaller dihedral angle (C2b−C1b−B1−C1), which are nearly parallel with an angle of 12.47(5)° between plane normals and an intramolecular π‐stacking interaction with interplanar distances of 3.1532(18) and 3.3724(19) Å. In the structure of the monoanion **1^.−^** (Figure 4), both carboranyls are rotated such that the C‐tolyl groups lie on the same side of the 3‐coordinate boron plane, whereas in the neutral compound one lies above and one below this plane. Similar to **1**, the configuration of the 3‐coordinate boron atom in **1^.−^** is trigonal planar with the sum of the C−B−C angles being 359.7(6)/360°, with slightly larger angles between the carboranes C1a−B1−C1b. However, the dihedral angle C2a−C1a−B1−C1 (94.4(2)/85.8°) is close to 90°, while C2b−C1b−B1−C1 (54.9(3)/46.0°) is smaller. In comparison to neutral **1**, the crystal structure of the paramagnetic monoanion **1^.−^** shows a large increase of the carborane C1a−C2a bond length by almost 0.6 Å resulting in bond cleavage (C1a−C2a=1.726(2)/1.708 Å in **1**; C1a−C2a=2.311(3)/2.284 Å in **1^.−^**, Table 1). This reduction‐induced cage opening is only observed in moiety **a**, and it is present in all 3 monoanion structures with different cations (Figures 4 and 5, Tables 1 and S2). A similar elongation of the C1−C2 bond in the carborane cage upon reduction was proposed by Weber, Fox and co‐workers from theoretical computations on the monoanion geometries of C‐diazaborolyl‐*o*‐carboranes and was attributed to the location of the negative charge in the cage.^[104]^ The bonds adjacent to C1a−C2a are also affected by the reduction, with a significant contraction of the B1−C1a (1.521(3)/1.528 Å) bond by ca. −0.09 Å compared to *d*(B1−C1a/C1b) of both carboranyl moieties in **1**, and to a smaller contraction (−0.03 Å) of the C2a−C3a (1.477(3)/1.475 Å) bond, which connects the tolyl moiety to carboranyl **a**. Radical anion **1^.−^** exhibits a small lengthening (+0.022 Å) of *d*(B1−C1) (1.575(3)/1.577 Å). The population of the π‐character B1−C1a LUMO orbital (see calculations) increases its bond order explaining the contraction and the tendency towards a 90° dihedral angle. The C1a−C2a σ*‐anti‐bonding orbital is also populated, lengthening the C1a−C2a bond. In contrast to the significant changes in the carboranyl moiety **a**, only small changes are observed for the carboranyl moiety **b** upon reduction, i. e., the C1b−C2b (1.732(3)/1.448 Å) cluster bond is slightly shorter by 0.03 Å in **1^.−^** than in **1**, as is the B1−C1b (1.616(3)/1.618 Å) bond by 0.01 Å, while *d*(C2b−C3b) (1.504(3)/1.501 Å) remains the same within 3 esd's. The slightly shorter bonds may be due to the absence of π‐stacking interactions between tolyl groups in **1^.−^** and, hence, not to the reduction process itself. This observation suggests that the *o*‐carboranyl moiety **b** plays no role in stabilizing the additional negative charge. The optimised geometry of the excited S_1_ state of **1** shows a strong resemblance to that of the radical anion **1^.−^** (Figure 5). A similarity of the S_1_ excited‐state and the monoanion geometries was proposed by Weber, Fox, and co‐workers for the C‐diazaborolyl‐*o*‐carborane from computations.^[104]^ Again, the *o*‐carboranyl group **b** and the 3‐coordinate B‐bound tolyl moiety of **1^.−^** are very similar to the neutral compound **1**. However, the parameters for the carboranyl moiety **a** show strong resemblance to the structure of **1^.−^**. The bond contraction of B1−C1a (1.522 Å), as well as the bond elongation of C1a−C2a (2.284 Å) in the S_1_ state agree well with the structural changes observed for **1^.−^**. The C2a−C3a (1.483 Å) bond is also significantly shorter than *d*(C2b−C3b) (1.504 Å). These similarities suggest comparable geometrical reorganisations during reduction and the CT process after excitation.

**Table 1:** Selected bond lengths [Å] and angles [°] of **1** and **{K[18]crown‐6 ⋅ (THF)_2_}^+^1^.−^** from experiment, optimised structures (Calc.), and optimised structure of the S_1_ state of **1** (S_1_‐Calc.).

Compound	1	1 Calc.^[a]^	1 S_1_‐ Calc.^[a]^	{K[18]crown‐6 ⋅ (THF)_2_}^+^1^.−^	1^.−^ Calc.^[a]^
B1−C1	1.553(3)	1.551	1.573	1.575(3)	1.577
B1−C1a	1.608(3)	1.610	1.522	1.521(3)	1.528
B1−C1b	1.626(2)	1.605	1.601	1.616(3)	1.618
C1a−C2a	1.726(2)	1.708	2.171	2.311(3)	2.284
C1b−C2b	1.761(2)	1.758	1.694	1.732(3)	1.748
C2a−C3a	1.507(2)	1.503	1.483	1.477(3)	1.475
C2b−C3b	1.502(2)	1.499	1.504	1.504(3)	1.501
∠C1−B1−C1a	118.4(1)	117.8	113.2	118.6(2)	119.1
∠C1−B1−C1b	120.2(1)	119.7	119.7	118.6(2)	118.4
∠C1a−B1−C1b	121.2(1)	122.4	127.0	122.2(2)	122.5
Sum ∠C−B1−C	359.8(3)	359.9	359.9	359.7(6)	360.0
∠C2a−C1a−B1−C1^[b]^	62.4(2)	65.1	73.6	94.4(2)	85.8
∠C2b−C1b−B1−C1^[b]^	−44.0(2)	−43.3	−31.5	54.9(3)	46.0

[a] B3LYP/6‐31G*. [b] Angles with respect to the 3‐coordinate borane plane.

### Photophysical properties

**Figure 6:**
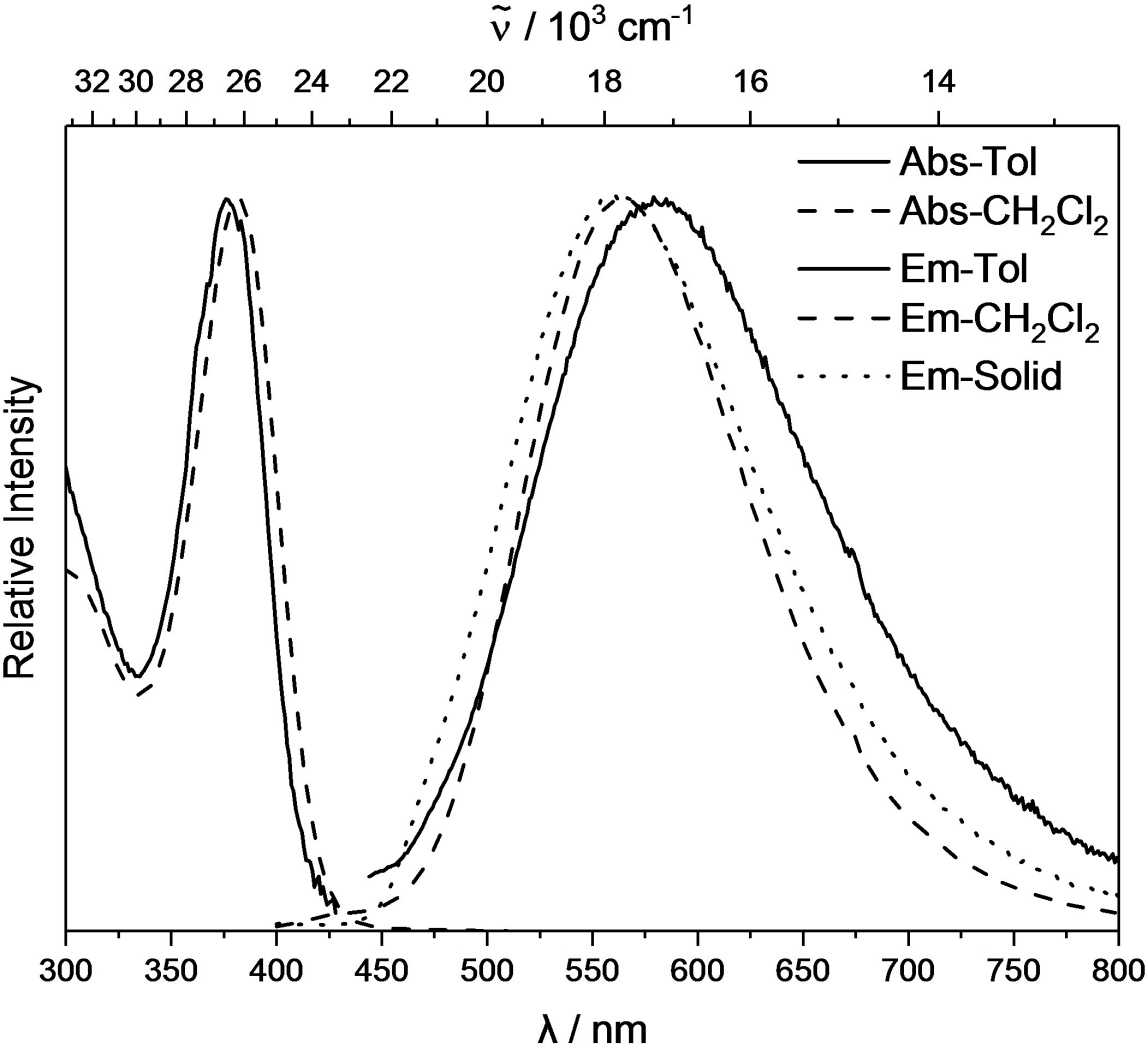
Absorption (<450 nm) and emission (>450 nm, excited at lowest energy absorption maximum) spectra of **1** in toluene (solid line), CH_2_Cl_2_ (dashed line) and solid state (dotted line).

**Figure 7:**
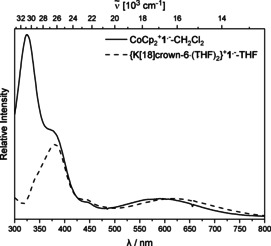
Absorption spectra of **CoCp_2_** ^**+**^ **1^.−^** (solid line) in CH_2_Cl_2_ and **{K[18]crown‐6 ⋅ (THF)_2_}^+^1^.−^** in THF (dashed line).

Photophysical data for **1** are listed in Table 2. The lowest energy absorption maximum of **1** shows almost no dependence on solvent polarity (toluene 376 nm, CH_2_Cl_2_ 381 nm) and is weakly allowed (ϵ=1800 M^−1^cm^−1^) (Figure 6). The excitation spectrum in the solid state exhibits a strong bathochromic shift (420 nm) of the lowest energy absorption maximum compared to that in solution. The emission band is strongly red shifted (561 nm as solid, 566 nm in CH_2_Cl_2_, 585 nm in toluene) compared to the absorption with large apparent Stokes shifts (6000 cm^−1^ as solid, 8600 cm^−1^ in CH_2_Cl_2_, 9500 cm^−1^ in toluene), indicating a significant geometry change in the excited state, typical of carborane chromophores.^[103]^ The emission maxima show a weak hypsochromic shift due to the direction change of the dipole moment from the ground to the excited state. The calculated orbital overlap parameter (Λ) of 0.42 at the CAM‐B3LYP level of theory suggests a CT from the initial excitation at the tolyl moiety to the *o*‐carborane cage (see TD‐DFT calculations).[^156^, ^157^] Quantum yields in solution were too low to be determined reliably, with decomposition visible in the absorption spectrum after extended measurements, but in the solid state, the quantum yield is 0.19, a behaviour typical of carborane chromophores.[^69^, ^84^, ^95^] Figure 7 displays the UV/Vis spectra of **1^.−^** in CH_2_Cl_2_ and THF, which are nearly identical, apart from a strong band at 323 nm, which can be ascribed to the cobaltocinium counter ion^[158]^ featuring a broad band in the low‐energy region (591 nm in CH_2_Cl_2_, 615 nm in THF).

**Table 2:** Photophysical data for compounds **1** and **1^.−^**.

Compound	Solvent	λ_abs_ [nm]	ϵ [M^−1^cm^−1^]	λ_em_ [nm]	Apparent Stokes Shift [cm^−1^]	τ [ns]	Φ_F_
1	Toluene	376		585	9500	‐^[a]^	‐^[a]^
1	CH_2_Cl_2_	381	1800	566	8600	‐^[a]^	‐^[a]^
1	Solid	420^[b]^		561	6000	14.0	0.19
CoCp_2_^+^1^.−^	CH_2_Cl_2_	591					
{K[18]crown‐6 ⋅ (THF)_2_}^+^1^.−^	THF	615

[a] Could not be determined reliably. [b] Calculated from excitation spectrum (see Supporting Information).

### DFT and TD‐DFT studies

**Figure 8:**
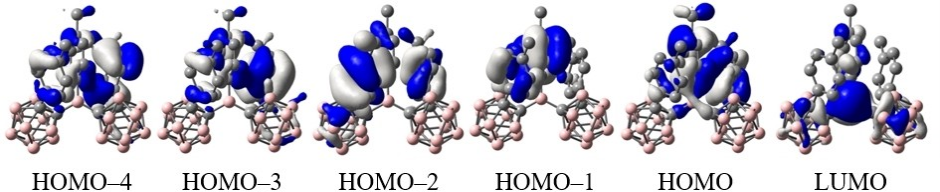
Frontier orbitals of **1**.

**Figure 9:**
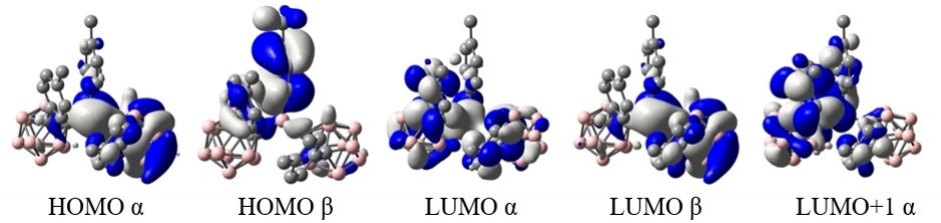
Frontier orbitals of **1^.−^**.

**Figure 10:**
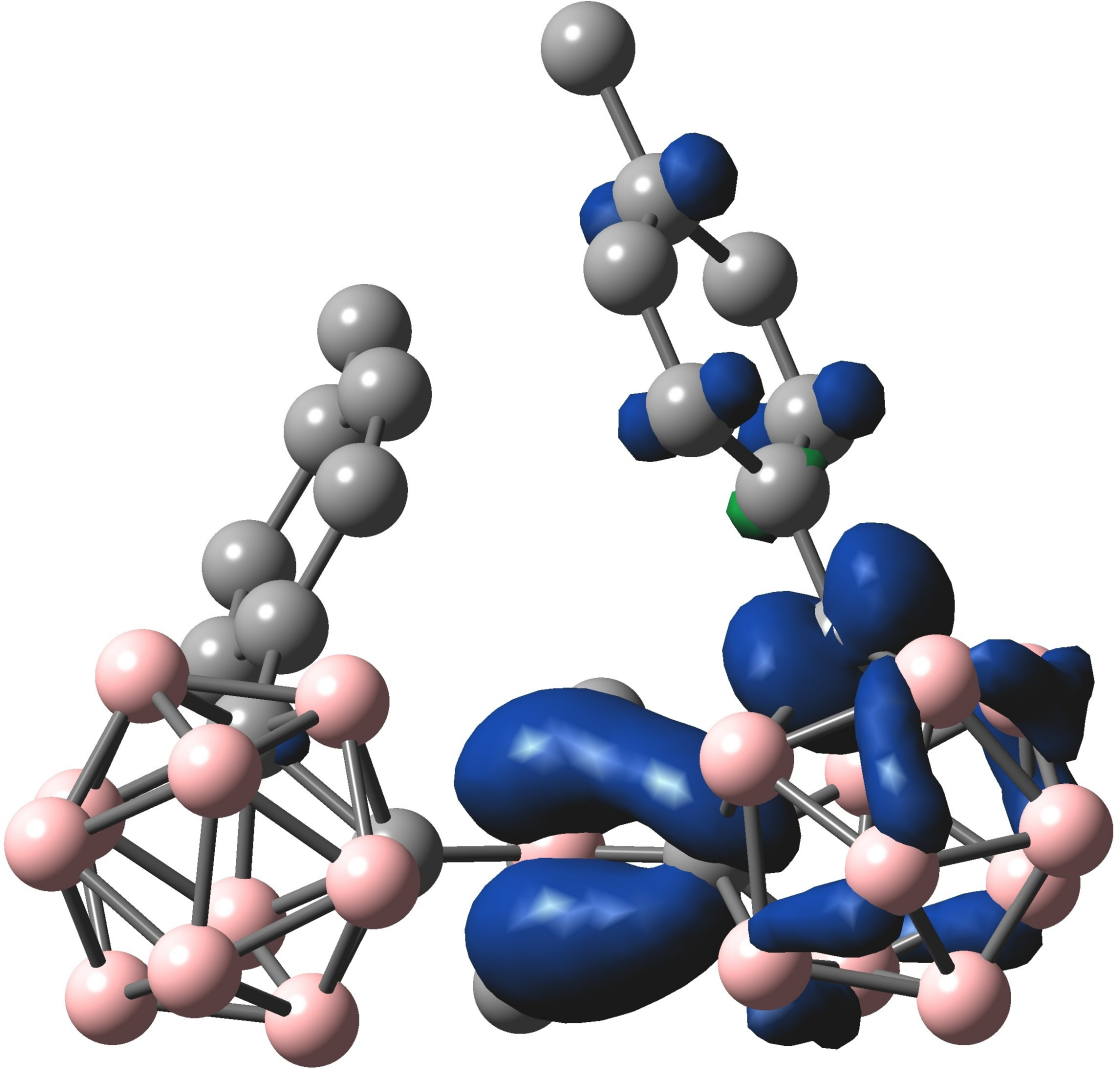
Spin density map of **1^.−^** at B3LYP/6‐31G* level of theory (ISO value=0.0025).

Optimised ground state structures were calculated at the B3LYP/6‐31G* level of theory in the gas phase starting from the solid state molecular geometries.[^103^, ^106^] For **1**, the HOMO is located on the tolyl moiety at the 3‐coordinate boron and on the carboranyl‐bound tolyl moiety **b** (Figure 8). All occupied orbitals down to HOMO‐4 are located on tolyl groups. The LUMO is located mostly on the 3‐coordinate boron with p_z_‐character and participation of the carborane C1−C2 σ*‐MO's depending on the respective dihedral overlap. In solution, with freely rotating carboranyl moieties, the first reduction, observed in the CV, should most likely take place at the 3‐coordinate boron p_z_ orbital followed by a geometrical reorganisation of one of the carborane cages, as has been described before.^[104]^ To gain insight into the photophysical properties of **1**, TD‐DFT calculations were carried out using the Coulomb attenuated functional CAM−B3LYP/6‐31G* (Table 3), which usually gives a better description of CT systems compared to B3LYP.^[159]^ The S_1_←S_0_ transition largely resembles a HOMO‐LUMO transition at 3.71 eV (λ=334 nm) that exhibits a relatively low oscillator strength of *f*=0.102 (Table 3). The use of the orbital overlap parameter Λ (0≤Λ≤1, where 0 and 1 correspond to no and complete overlap, respectively) offers a method to quantify the spatial overlap of transition orbitals.^[156]^ With a relatively high separation given by Λ=0.42 together with the observed spectrum, the first transition can be categorized as CT. The simulated data are in good agreement with the UV/Vis spectrum (see Supporting Information). For **1^.−^**, the HOMO α (SOMO) is mainly delocalised over the carboranyl moiety **a** (C1−C2 σ*‐anti‐bonding orbital) with some contribution from the aligned 3‐coordinate boron atom p_z_‐orbital. The LUMO α is spread over the carboranyl moiety **b**, the *o*‐carborane‐bound tolyl moieties **a** and **b**, and the 3‐coordinate boron (Figure 9). The S_1_←S_0_ transition between these two MO's has LE character (Λ=0.68) and a small energy gap of 2.08 eV (λ=595.9 nm) with an oscillator strength of 0.1301 (Table 4), agreeing nicely with the absorption spectrum (see Supporting Information). Given the participation of the carboranyl moiety **b** in the first transition, alternating carborane radicals seem plausible. A continuous delocalisation between both appears to be inhibited by the energy necessary to reorganize both carboranyl moieties simultaneously. The spin density map of **1^.−^** at the B3LYP/6‐31G* level of theory reveals a distribution of 57 % on the carborane cage **a**, 37 % on B1 and 5 % on the carboranyl‐bound tolyl **a** (Figure 10). This distribution underlines the significant delocalisation of the negative charge between B1, C1a, and C2a and provides confirmation of the hypothesis derived from the crystal structure, suggesting that *o*‐carborane is a stronger acceptor than the borane unit, confirming previous reports.^[106]^

**Table 3:** TD‐DFT calculated transitions (toluene) of **1** (CAM‐B3LYP/6‐31G*).

FC‐S_n_	E[eV] (E[nm])	f	Contribution >10 %	Λ
S_1_	3.71 (334)	0.102	HOMO ‐> LUMO (86 %)	0.42
S_2_	4.27 (290)	0.004	H‐2 ‐> LUMO (12 %) H‐1 ‐> LUMO (76 %)	0.33
S_3_	4.53 (274)	0.013	H‐2 ‐> LUMO (82 %) H‐1 ‐> LUMO (13 %)	0.32
S_4_	4.61 (269)	0.014	H‐4 ‐> LUMO (11 %) H‐3 ‐> LUMO (73 %)	0.36
S_5_	4.96 (250)	0.006	H‐4 ‐> LUMO (76 %) H‐3 ‐> LUMO (10 %)	0.30

**Table 4:** TD‐DFT calculated transitions (gas phase) of **1^.−^** (B3LYP/6‐31G*).

FC‐S_n_	E[eV] (E[nm])	f	Contribution >10 %	Λ
S_1_	2.08 (596)	0.1301	HOMO α ‐> LUMO α (92 %)	0.68
S_2_	2.35 (528)	0.0054	HOMO α ‐> L+1 α (93 %)	0.54
S_3_	2.79 (443)	0.0014	HOMO α ‐> L+2 α (71 %) HOMO α ‐> L+3 α (27 %)	0.50
S_4_	2.88 (430)	0.003	HOMO α ‐> L+2 α (27 %) HOMO α ‐> L+3 α (71 %)	0.68
S_5_	3.13 (396)	0.0042	HOMO α ‐> L+4 α (35 %) HOMO β ‐> LUMO β (46 %)	0.54

## Conclusions

We report the synthesis and properties of an unusual bis(*o*‐carboranyl) substituted 3‐coordinate borane [(1‐(4‐MeC_6_H_4_)‐*closo*‐1,2‐C_2_B_10_H_10_‐2‐)_2_(4‐MeC_6_H_4_)B] (**1**). Cyclic voltammetry studies revealed a partially reversible one‐electron first reduction, and the resulting radical anion **1^.−^** was isolated using different reduction protocols. EPR spectroscopy confirmed the paramagnetic nature of the anion and the involvement of the *o*‐carboranyls in the delocalisation of the unpaired electron spin. The solid‐state structure of the radical anion revealed major contributions of B1 and one of the *o*‐carboranyl moieties to the delocalisation of the extra electron, with negligible change of bond distances within the second *o*‐carboranyl moiety. The perpendicular orientation of the reduced *o*‐carborane fragment facilitates optimal overlap between the 3‐coordinate boron p_z_‐orbital and the C1−C2 σ*‐anti‐bonding orbital leading to a shorter B1−C1a and longer C1a−C2a bond. Strong similarities between the 2n+3 SE *o*‐carborane structure of the radical anion **1^.−^** and the optimised structure of the S_1_ state of **1** confirm predictions regarding geometrical reorganisation in the CT process. The photophysical studies show a CT emission with a large Stokes shift of up to 9500 cm^−1^. Like other known carborane dyads, **1** has a small quantum yield in solution with a strong increase in the solid state (Φ_F_=19 %). Upon reduction, a broad low energy absorption appears at ca. 600 nm. The experimental observations are in good agreement with the calculations at the CAM‐B3LYP/6‐31G* level of theory.

## Conflict of interest

The authors declare no conflict of interest.

## Supporting information

As a service to our authors and readers, this journal provides supporting information supplied by the authors. Such materials are peer reviewed and may be re‐organized for online delivery, but are not copy‐edited or typeset. Technical support issues arising from supporting information (other than missing files) should be addressed to the authors.

Supplementary
