# Supplementary material for: Synthesis and Structure of an o‐Carboranyl‐Substituted Three‐Coordinate Borane Radical Anion
Source: Chemistry. 2021 May 3;27(31):8159–67. doi: 10.1002/chem.202100938 (PMC8252506; doi:10.1002/chem.202100938)
Supplement: Supplementary file 1 — Supplementary [file CHEM-27-8159-s001.pdf]

Chemistry–A European Journal

Supporting Information

Synthesis and Structure of an *o*-Carboranyl-Substituted Three-Coordinate Borane Radical Anion

Johannes Krebs, Martin Haehnel, Ivo Krummenacher, Alexandra Friedrich, Holger Braunschweig, Maik Finze, Lei Ji, and Todd B. Marder*

Supporting Information

Table of Contents

General experimental details S2
Synthetic procedures S6
EPR measurement S10
Single-crystal X-ray diffraction S11
Electrochemistry S15
Photophysical properties S16
TD-DFT calculations S18

General experimental details

Unless otherwise noted, the following conditions apply.

All syntheses were carried out using standard Schlenk and glovebox techniques under an argon atmosphere. The solvents used were dried using a solvent purification system (SPS) from Innovative Technology and all except CH_2Cl_2 (DCM) were degassed and stored over sodium-potassium-alloy. DCM was passed through alumina and stored over activated molecular sieves. Deuterated solvents (CD_2Cl_2 and C_6D_6) used for NMR spectroscopy were purchased from Cambridge Isotope Laboratories. C_6D_6 was dried over molecular sieves and stored under an argon atmosphere before use. *n*-Butyllithium (2.5 M solution in hexane) was purchased from Acros Organics and used as received. $CoCp_2$ was kindly provided by Prof. Dr. Udo Radius of our Institute. The compound 1-1-(4-methylphenyl)-1,2-dicarba-*closo*-dodecarborane was prepared according to a literature procedure.[1] All other starting materials were purchased from commercial sources and were used without further purification.

NMR: Spectra were recorded on a Bruker Avance 500 FT NMR spectrometer (operating at ^{1}H: 500 MHz, $^{11}B\{^{1}H\}$: 160 MHz, $^{13}C\{^{1}H\}$: 126 MHz. Chemical shifts (δ) are given in ppm and are referenced to external $BF_3{\cdot}Et_2O$ ($^{11}B\{^{1}H\}$). ^{1}H NMR spectra were referenced via residual proton resonances of CD_2Cl_2 (5.32 ppm) and C_6D_6 (7.16 ppm).[2] $^{13}C\{^{1}H\}$ spectra were referenced to CD_2Cl_2 (53.84 ppm) and C_6D_6 (128.06 ppm).[2] GCMS analyses were performed on an Agilent Technologies GCMS system (GC 7890A, EI-MS 5975C). HRMS were recorded using a Thermo Scientific Exactive Plus Orbitrap MS system with either an Atmospheric Sample Analysis Probe (ASAP) or by Electro-Spray Ionization (ESI).

Single-crystal X-ray diffraction: Crystals suitable for single-crystal X-ray diffraction were selected, coated in perfluoropolyether oil or polybutyl oil, mounted on a polyimide microloop (MicroMounts fromMiTeGen) and transferred to a stream of cold nitrogen (Oxford Cryostream 700 or 800, respectively). Due to the lack of transparency for some samples, the crystal choice was based on its shape. As these crystals were extremely unstable in air, they were rapidly mounted under an argon stream and cooled at 100 K using an Oxford Cryostream low-temperature device attached to the diffractometer. Diffraction data of **1** and $\mathbf{\{Na{\cdot}(THF)_6\}^{+}1^{\bullet-}}$ were collected on a Bruker X8 Apex II 4-circle diffractometer with a CCD area detector, using Mo-Kα radiation generated by a Nonius FR591 rotating anode and monochromated by multi-layer focusing mirrors.

Diffraction data of **1**$^{\bullet-}$ and **CoCp$_2$$^+1^{\bullet-}$** were collected on a Rigaku Oxford Diffraction XtaLAB Synergy diffractometer with a semiconductor HPA-detector (HyPix-6000) and multi-layer mirror monochromated Cu-K$_\alpha$ radiation at 100 K.

The images were processed and corrected for Lorentz-polarization effects and absorption (empirical scaling) as implemented in the Bruker software packages (**1**, **{Na·(THF)$_6$}$^+$1$^{\bullet-}$**) or using the CrysAlis^{Pro} software from Rigaku Oxford Diffraction (**1**$^{\bullet-}$, **CoCp$_2$$^+1^{\bullet-}$**). The structures were solved using the intrinsic phasing method (SHELXT)[3] and Fourier expansion technique. All non-hydrogen atoms were refined in anisotropic approximation, with all hydrogen atoms 'riding' in idealized positions, by full-matrix least squares against F^2 of all data, using SHELXL[4] software and the SHELXLE[5] graphical user interface. Due to strong disorder in the crystal structures of **{Na·(THF)$_6$}$^+$1$^{\bullet-}$** and **CoCp$_2$$^+1^{\bullet-}$**, reflection intensities were very weak, especially for high-resolution data. Hence, the data:parameter ratio was very low resulting in a reduced quality of the diffraction data and structure refinements. The data collected on **CoCp$_2$$^+1^{\bullet-}$** suffer from systematic errors in absorption correction and Co fluorescence scattering due to the choice of a very large plate-like crystal. Furthermore, the unit cell of **CoCp$_2$$^+1^{\bullet-}$** contains disordered tetrahydrofuran solvent molecules, several of which have been treated as a diffuse contribution to the overall scattering without specific atom positions by using SQUEEZE/PLATON.[6] This can introduce further systematic errors. Nevertheless, the crystal structures and, hence, molecular geometries could be solved from these data and crystal structures are included in the SI as a proof of conformation. Diamond[7] software was used for graphical representation. Crystal data and experimental details are listed in Table 1; full structural information has been deposited with the Cambridge Crystallographic Data Centre. CCDC-2040247 (**1**), 2040248 (**1**$^{\bullet-}$) and 2043596 (**{Na·(THF)$_6$}$^+$1$^{\bullet-}$**).

Photophysical measurements: All measurements were performed in standard quartz cuvettes (1 cm x 1 cm cross-section). UV–visible absorption spectra were recorded using an Agilent 8453 diode array UV-visible spectrophotometer.

Emission spectra were recorded using an Edinburgh Instruments FLSP920 spectrometer equipped with a double monochromator for both excitation and emission, operating in right-angle geometry mode, and all spectra were fully corrected for the spectral response of the instrument. All solutions used for photophysical measurements had a concentration lower than 2×10^{-5} M to minimize inner filter effects during fluorescence measurements.

Fluorescence quantum yields were measured using a calibrated integrating sphere (inner diameter: 150 mm) from Edinburgh Instruments combined with the FLSP920 spectrometer

described above. For solution-state and solid-state measurements, the longest-wavelength absorption maximum of the compound in the respective solvent was chosen as the excitation wavelength.

Fluorescence lifetimes were recorded using the time-correlated single-photon counting (TCSPC) method using the same FLSP920 spectrometer described above. Solutions were excited with a picosecond pulsed diode laser at an emission maximum of 376.6 nm. The full width at half maximum (FWHM) of the laser pulses were ca. 72 ps, while the instrument response function (IRF) had a FWHM of ca. 1.0 ns, measured from the scatter of a Ludox solution at the excitation wavelength. Decays were recorded to at least 10000 counts in the peak channel with a record length of at least 1000 channels. The band pass of the monochromator was adjusted to give a signal count rate of <10 kHz. Iterative reconvolution of the IRF with one decay function and non-linear least-squares analysis were used to analyse the data. The quality of the fit was judged by the calculated value of the reduced χ^2 and visual inspection of the weighted residuals.

Electrochemical measurements: All cyclic voltammetry experiments were conducted in an argon-filled glovebox using a Gamry Instruments Reference 600 potentiostat. A standard three-electrode cell configuration was employed using a platinum disk working electrode, a platinum wire counter electrode, and a silver wire reference electrode separated by a Vycor frit, serving as the reference electrode. The redox potentials are referenced to the ferrocene/ferrocenium ([Fc/Fc^+]) redox couple) as an internal standard. Tetra-*n*-butylammonium hexafluorophosphate ([$n$$Bu_4N$][$PF_6$]) was employed as the supporting electrolyte.

EPR measurements: Measurements at X-band (9.85 GHz and 9.38 GHz) were carried out at room temperature and 240 K using a Bruker ELEXSYS E580 CW EPR spectrometer. CW EPR spectra were measured using 1 mW microwave power and 1 G field modulation at 100 kHz, with a conversion time of 20 ms.

Theoretical Studies: All calculations (DFT and TD-DFT) were carried out with the Gaussian 09 (9.E.01)[8] program package and were performed on a parallel cluster system. GaussView (6.0.16) and multiwfn[9] were used to visualize the results, to measure calculated structural parameters, and to plot orbital surfaces (isovalue: ± 0.030 [e a_0^{-3}]$^{1/2}$). The ground-state geometries were optimized using the B3LYP functional[10] in combination with the 6-31+G(d) basis set.[11,12] The D3 dispersion correction of Grimme and coworkers was used.[13] The

ultrafine integration grid and symmetry constraints were used for all molecules. Frequency calculations were performed on the optimized structures to confirm them to be local minima showing no negative (imaginary) frequencies. Based on these optimized structures, the lowest-energy vertical transitions (using the polarizable continuum model) were calculated (singlets, 25 states) by TD-DFT, using the Coulomb attenuated functional CAM-B3LYP[14] as well as B3LYP. The CAM-B3LYP functional has been shown to more accurately describe CT systems in comparison to B3LYP.[15] The optimized ground-state geometries were used as starting coordinates for TD-DFT geometry optimizations. The S_1 state of **1** were optimized using 10 excited states with the B3LYP functional in combination with the 6-31g* basis set and D3 dispersion correction and no symmetry constraints.

Synthetic procedures

Synthesis of bis(1-(4-tolyl)-carboran-2-yl)-(4-tolyl)-borane (1)

The compound 1-(4-methylphenyl)-1,2-dicarba-*closo*-dodecarborane (500 mg, 1.91 mmol) was dissolved in toluene (15 mL) and a 2.5 M *n*BuLi solution in hexane (0.40 mL, 1.04 mmol) was added dropwise at –78 °C. The reaction mixture was slowly warmed to room temperature and stirred at 80 °C overnight. Then, the reaction was cooled to –78 °C and dibromo(4-methylphenyl)borane (940 mg, 4.01 mmol) in toluene (5 mL) was added dropwise after which the reaction was slowly warmed to room temperature and stirred at room temperature for 4 d. The suspension was filtered, washed with cold toluene (5 mL), diethyl ether (10 mL), and hexane (10 mL). The crude product was recrystallized from toluene to give [(1-(4-MeC_6H_4)-*closo*-1,2-$C_2B_{10}H_9$-2-)$_2$(4-MeC_6H_4)B] (**1**) (434 mg, 0.76 mmol) as a pale yellow solid in 40% yield. Traces of 1-(4-methylphenyl)-1,2-dicarba-*closo*-dodecarborane and 4-methylphenyl-boronicacid can be observed in the ^{1}H, ^{11}B and ^{13}C NMR spectra due to the instability of the compound in solution.

^{1}H NMR (500 MHz. C_6D_6, ppm): δ 6.73 (d, J = 8 Hz, 4H), 6.37 (d, J = 8 Hz, 4H), 6.18 (d, J = 8 Hz, 2H), 5.49 (d, J = 8 Hz, 2H), 4.5–2.0 (br, 20H), 1.95 (s, 3H), 1.90 ppm (s, 6H).
^{11}B NMR (160 MHz. C_6D_6, ppm): δ 71.5, 5.3, –2.4, –7.2, –9.6.
^{11}B{^{1}H} NMR (160 MHz. C_6D_6, ppm): δ 71.5, 5.3, –2.5, –7.2, –9.8.
^{13}C{^{1}H} NMR (125 MHz, C_6D_6, ppm): δ 141.3, 139.6, 139.0, 131.3, 130.1, 129.7, 128.9, 126.4, 88.9, 81.4, 21.9, 21.3.
HR-MS (ASAP^{+}) (*m/z*): calc. for $[C_{25}H_{41}B_{21}]^{+}$ 568.53022; found 568.52916 $[M+H]^{+}$.
Elem. Anal. Calc. (%) for $C_{25}H_{41}B_{21}$: C 52.81, H 7.27; found: C 52.90, H 7.36.

Preparation of {K[18]crown-6·(THF)$_2$}$^{+}$1$^{•–}$

In an argon filled glove box, compound **1** (5 mg, 9 μmol) was dissolved in THF (1 mL) and 88 μL of a 0.1 M K[18]crown-6 naphthalenide stock solution in THF was added. A deep blue colour could be observed immediately. Dry hexane vapor was diffused at −30 °C into 1 mL GC vials containing the reaction solution. Dark blue crystalline solid formed after 1-2 weeks giving **{K[18]crown-6·(THF)$_2$}$^{+}$1$^{•–}$** (8 mg, 7 μmol) in 84% yield.

Due to insufficient amounts of isolated compound, it was only characterized by single-crystal X-ray diffraction.

Preparation of $CoCp_2^+1^{\bullet-}$

In an argon filled glove box, compound **1** (5 mg, 9 μmol) and $CoCp_2$ (2 mg, 11 μmol) were dissolved in CH_2Cl_2 (1 mL) and the reaction turned deep blue immediately. Hexane (5 mL) was added and a solid precipitated at −30 °C overnight which was washed with hexane and toluene several times giving **$CoCp_2^+1^{\bullet-}$** (6 mg, 8 μmol) as a dark blue solid in 90% yield.

Due to insufficient amounts of isolated compound, it was only characterized by single-crystal X-ray diffraction.

Preparation of $\{Na{\cdot}(THF)_6\}^+1^{\bullet-}$

In an argon filled glove box, compound **1** (5 mg, 9 μmol) was dissolved in THF (1 mL) and a grain of cleanly cut sodium was added. The reaction turned deep blue immediately. From the filtered solution, crystallization was achieved by hexane diffusion at −30 °C. Only a small of **$\{Na{\cdot}(THF)_6\}^+1^{\bullet-}$** was obtained and used for single-crystal structure analysis.

.

^{1}H NMR spectrum of **1** in C_6D_6

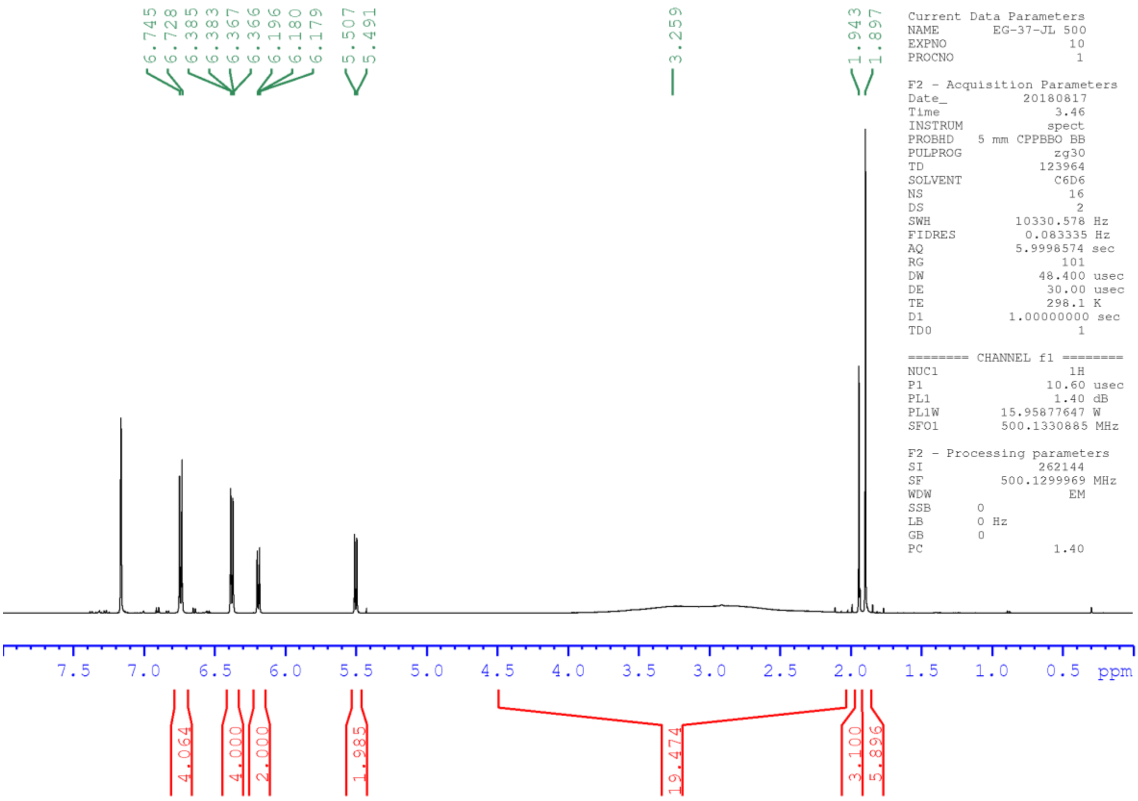

^{11}B NMR spectrum of **1** in C_6D_6

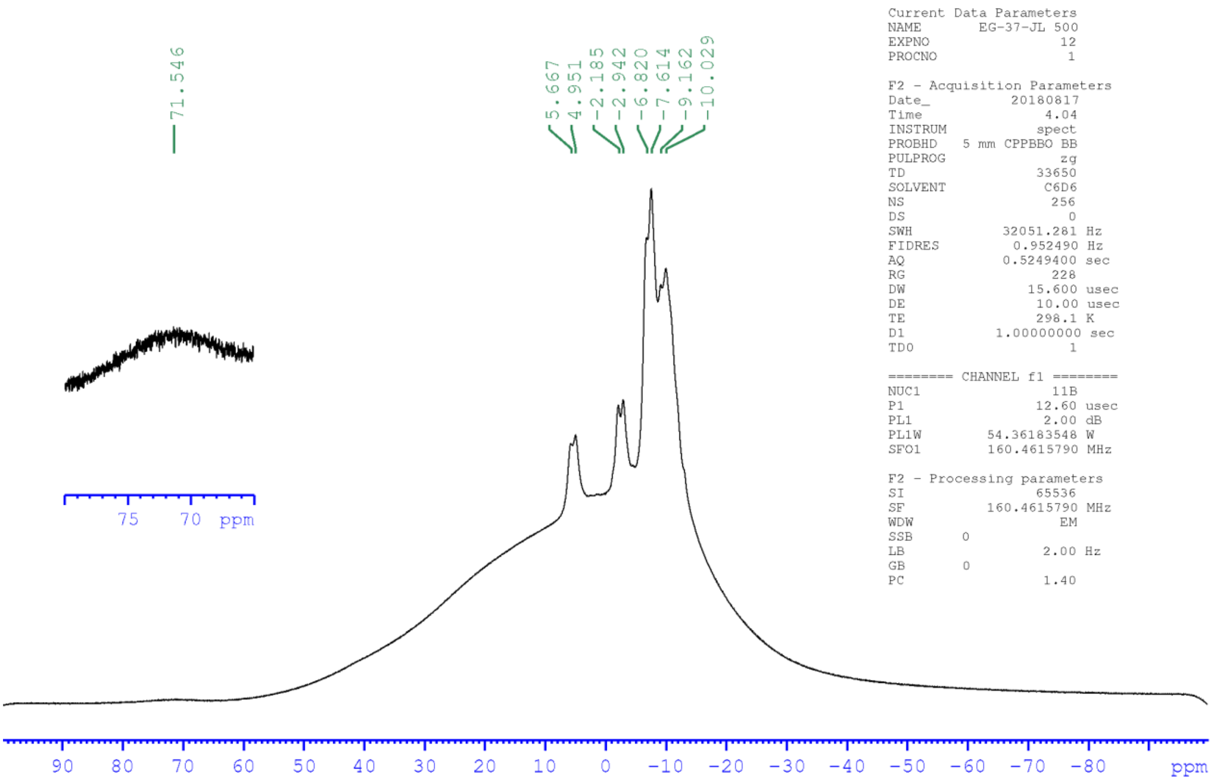

$^{11}B\{^1H\}$ NMR spectrum of 1 in C_6D_6

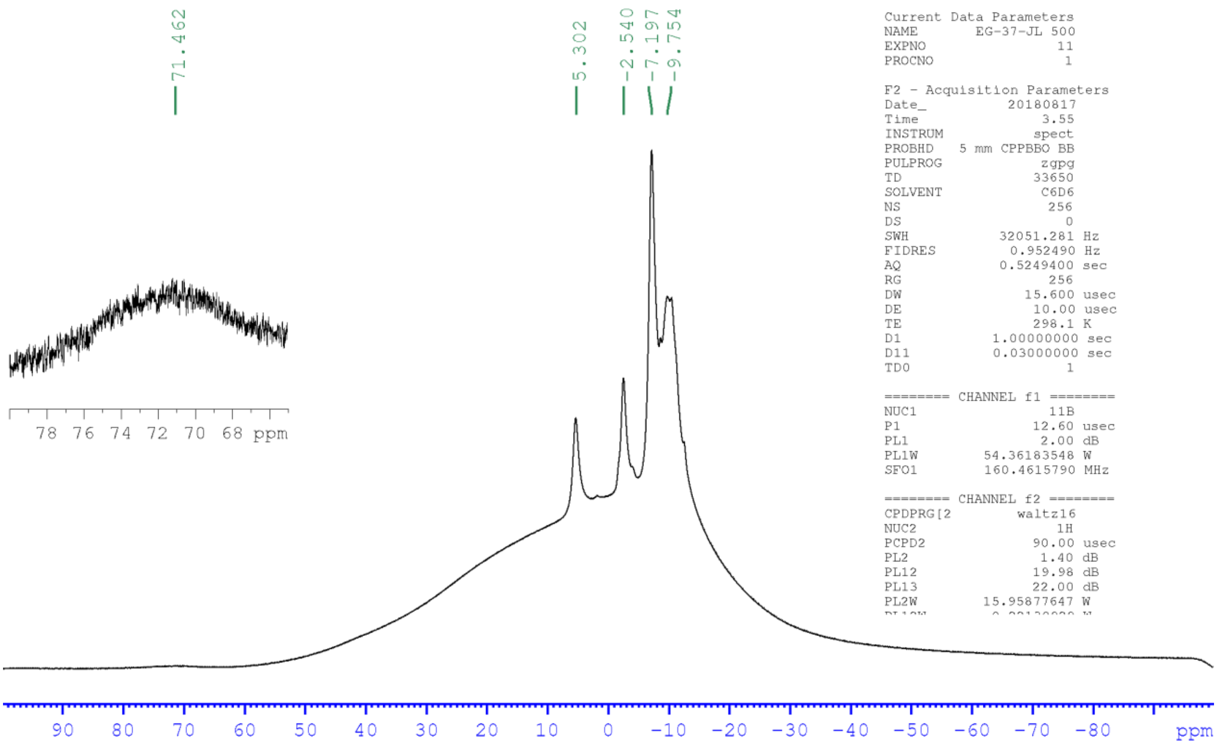

$^{13}C\{^1H\}$ NMR spectrum of 1 in C_6D_6

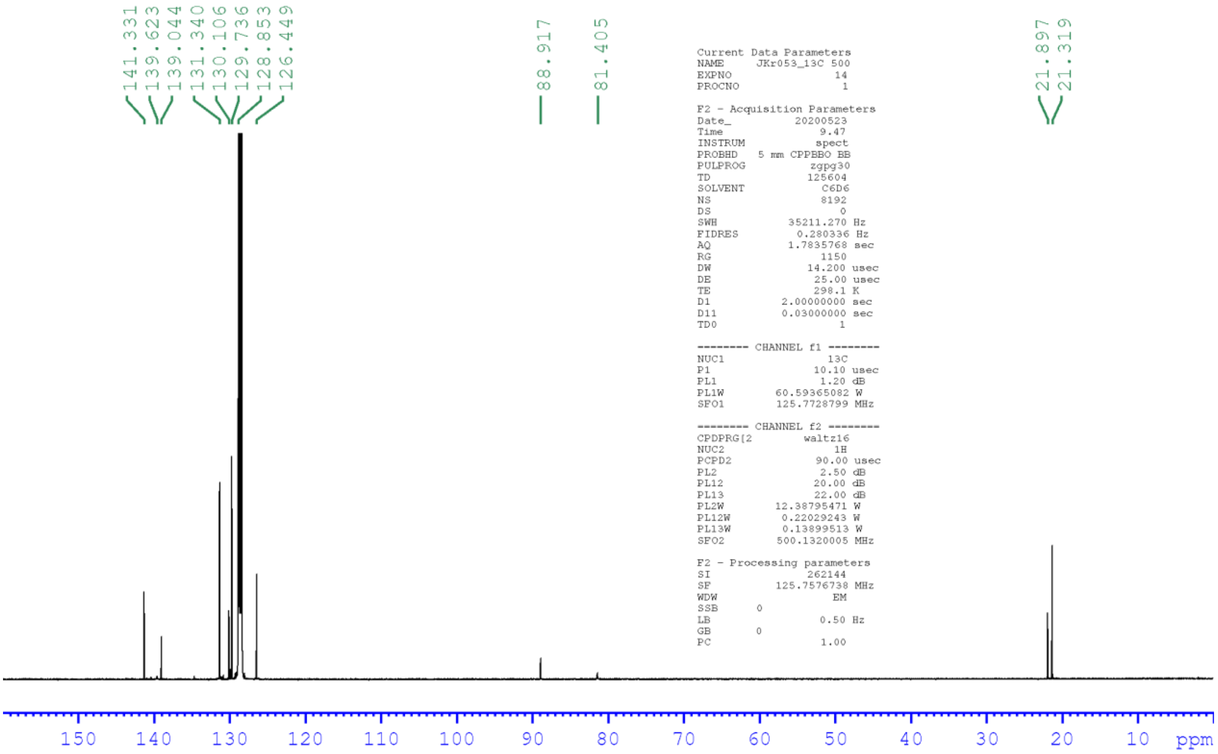

EPR measurement

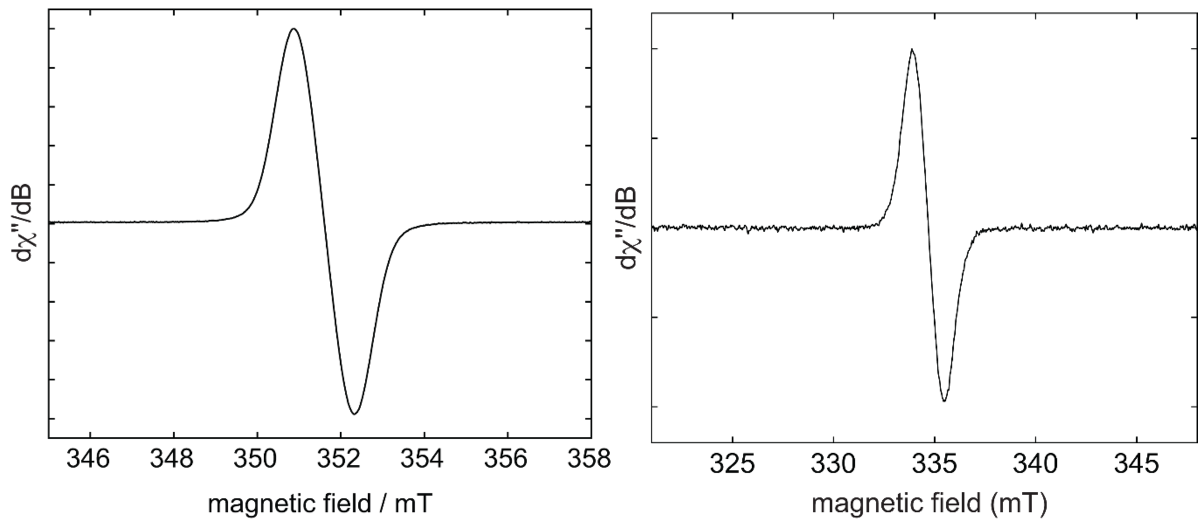

Figure S1. *Left*: Room temperature continuous-wave (CW) X-band (9.85 GHz) EPR spectrum of $\mathbf{Na^{+}1^{\bullet-}}$ in tetrahydrofuran. The isotropic *g* value is 2.0026 and the peak-to-peak linewidth is 1.4 mT. *Right*: The signal shape remains unchanged upon cooling to 240 K (9.38 GHz).

Single-crystal X-ray diffraction

Table S1. Details on single-crystal X-ray diffraction data and structure refinements of **1**, **1$^{•−}$**, **CoCp$_2$$^+1^{•−}$**, and **{Na·(THF)$_6$}$^+1^{•−}$**.

Data	**1**	**1$^{•−}$**	**CoCp$_2$$^+1^{•−}$**	**{Na·(THF)$_6$}$^+$1$^{•−}$**
CCDC	2040247	2040248		2043596
Empirical formula	$C_{25}H_{41}B_{21}$	$C_{25}H_{41}B_{21}$ · $C_{20}H_{40}KO_8$ · C_4H_8O	$C_{25}H_{41}B_{21}$· $C_{10}H_{10}Co$ · 0.75(C_4H_8O) [+solvent]	$C_{25}H_{41}B_{21}$ · 6(C_4H_8O)Na
ρ_{calc}/g·cm^{-3}	1.146	1.178	1.038	1.132
F(000)	1184	2316	3384	4376
Crystal size/mm^3	0.35×0.25×0.20	0.22×0.04×0.03	0.79×0.44×0.07	0.17×0.11×0.05
Crystal color, habit	colourless block	Blue block	blue plate	blue plate
μ/mm^{-1}	0.055	1.138	2.768	0.071
M_r/g·mol^{-1}	568.59	1088.31	3247.09 [+solvent]	1024.20
Temperature/K	100(2)	100(2)	100(2)	100(2)
Radiation, λ/Å	MoK$_\alpha$, 0.71073	CuK$_\alpha$, 1.54184	CuK$_\alpha$, 1.54184	MoK$_\alpha$, 0.71073
Crystal system	monoclinic	monoclinic	triclinic	Monoclinic
Space group	$P2_1/n$	$P2_1$/n	$P\bar{1}$	$P2_1$/n
a/Å	10.777(6)	10.4239(2)	12.81360(10)	13.689(13)
b/Å	25.213(13)	37.4152(6)	29.3642(4)	46.23(4)
c/Å	12.356(7)	15.7574(3)	29.8382(3)	19.737(19)
α/°	90	90	69.5130(10)	90
β/°	101.036(8)	92.772(2)	81.3770(10)	105.82(2)
γ/°	90	90	84.9700(10)	90
Volume/Å^3	3295(3)	6138.39(19)	10390.5(2)	12017(20)
Z	4	4	2	8
2Θ/°	3.23–53.47	4.724–155.044	5.244–136.50	2.776–50.246
Unique reflections	7001	12733	36295	21468
Parameters / restraints	438/0	842/361	2382 / 886	1597 / 690
GooF on F^2	1.026	1.035	1.554	0.998
R_1 [I>=2σ (I)]	0.0480	0.0602	0.1214	0.1016
wR^2 [all data]	0.1255	0.1690	0.3754	0.2766
Max./min. residual electron density/ e Å^{-3}	0.30 / –0.26	0.82 / –0.55	3.82 / –0.90	0.58 / –0.43

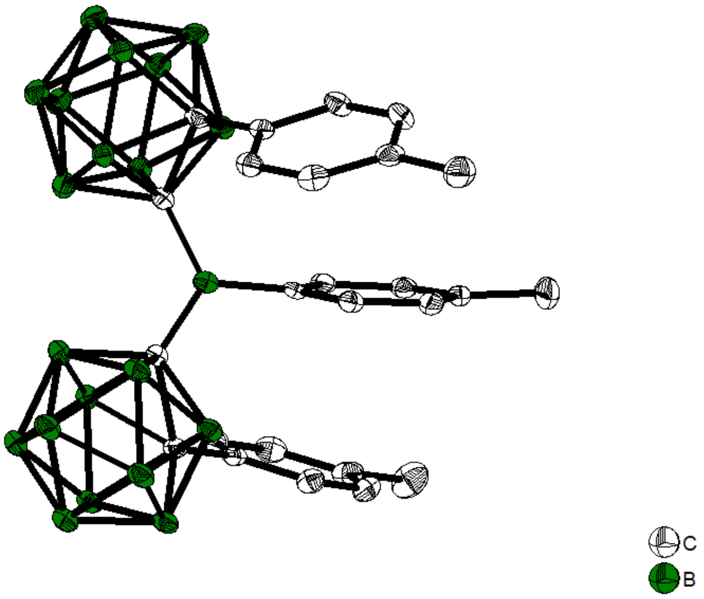

Figure S2: Solid state molecular structure of **1** from single-crystal X-ray diffraction at 100 K. Atomic displacement ellipsoids are drawn at the 50% probability level and hydrogen atoms are omitted for clarity.

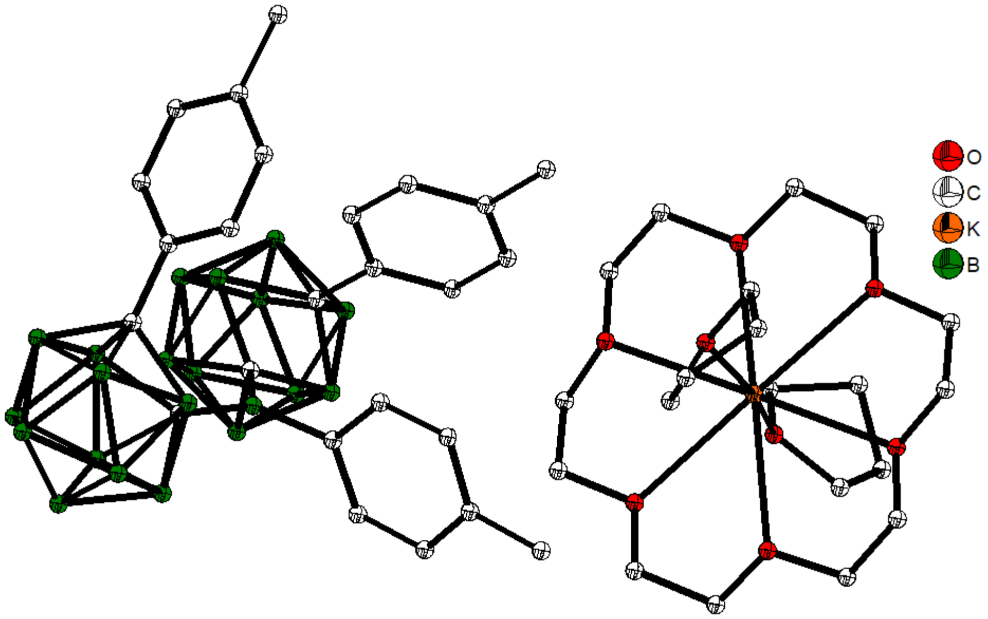

Figure S3: Solid state molecular structure of **{K[18]crown-6·(THF)$_2$}$^+$1$^{\bullet-}$** from single-crystal X-ray diffraction at 100 K. Atomic displacement ellipsoids are drawn at the 50% probability level and solvent molecules and hydrogen atoms are omitted for clarity.

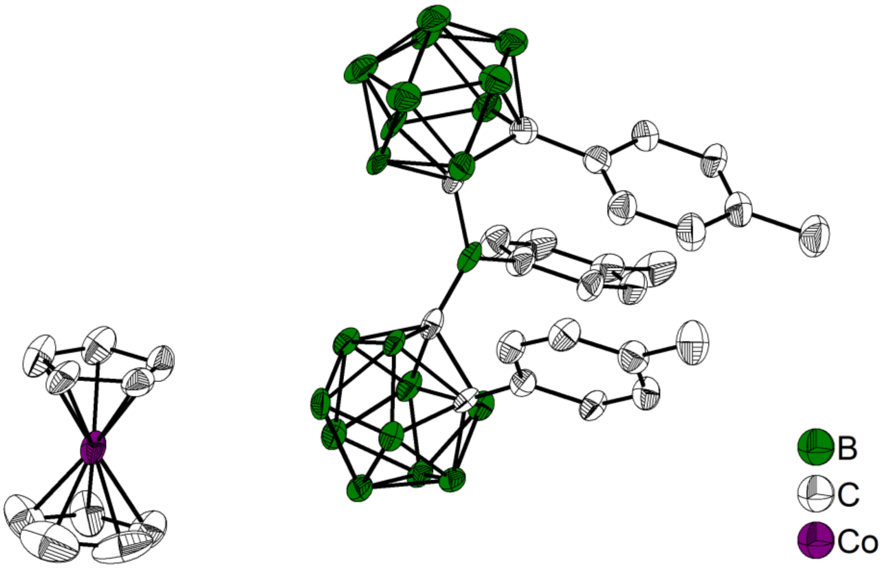

Figure S4: Solid state molecular structure of **$CoCp_2^{+}$1$^{\bullet-}$** from single-crystal X-ray diffraction at 100 K. Atomic displacement ellipsoids are drawn at the 50% probability level and hydrogen atoms as well as solvent molecules are omitted for clarity. Only one of four symmetry-independent anions and cations each are shown.

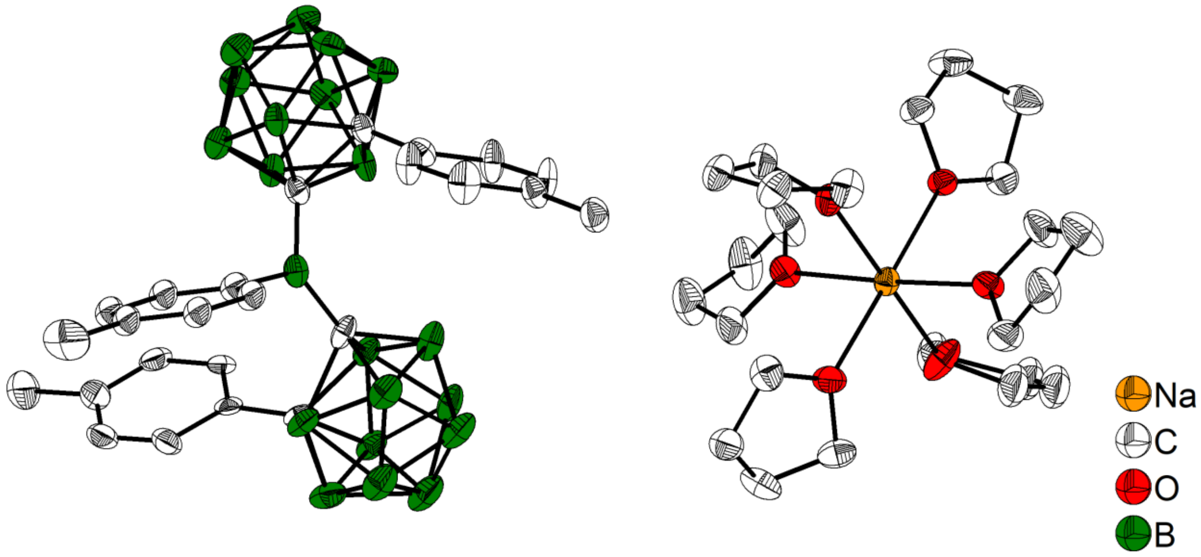

Figure S5: Solid state molecular structure of **$\{Na\cdot(THF)_6\}^{+}$1$^{\bullet-}$** from single-crystal X-ray diffraction at 100 K. Atomic displacement ellipsoids are drawn at the 50% probability level, and hydrogen atoms as well as the minor occupied parts of disordered THF and tolyl groups are omitted for clarity. Only one of two symmetry-independent anions and cations each are shown.

Table S2: Selected bond lengths [Å] and angles [°] for **1** and **{K[18]crown-6·(THF)$_2$}$^+$1$^{•-}$** (abbreviated as **1$^{•-}$** in the table), **CoCp$_2$$^+1^{•-}$**, and **{Na·(THF)$_6$}$^+1^{•-}$** in the solid state at 100 K.

Compound	**1**	**1$^{•-}$**	**CoCp$_2$$^+1^{•-}$**	**{Na·(THF)$_6$}$^+$1$^{•-}$**
B1–C1	1.553(3)	1.575(3)	1.566(8) / 1.576(8) / 1.576(7) / 1.564(7)	1.580(8) / 1.604(10) and 1.610(10)
B1–C1a	1.608(3)	1.521(3)	1.536(7) / 1.527(7) / 1.525(8) / 1.532(8)	1.495(10) / 1.519(10)
B1–C1b	1.626(2)	1.616(3)	1.595(8) / 1.604(8) / 1.613(8) / 1.613(8)	1.625(10) / 1.610(10)
C1a–C2a	1.726(2)	2.311(3)	2.318(9) / 2.336(8) / 2.334(8) / 2.319(9)	2.335(8) / 2.339(8)
C1b–C2b	1.761(2)	1.732(3)	1.729(7) / 1.727(7) / 1.730(6) / 1.715(6)	1.739(8) / 1.742(8)
C2a–C3a	1.507(2)	1.477(3)	1.468(11) / 1.485(9) / 1.465(6) / 1.458(6)	1.477(9) / 1.476(7)
C2b–C3b	1.502(2)	1.504(3)	1.503(9) / 1.488(9) / 1.490(8) / 1.491(8)	1.502(7) / 1.504(7)
∠ C1–B1–C1a	118.4(1)	118.6(2)	119.4(5) / 119.0(5) / 119.3(5) / 118.9(4)	118.4(5) / 119.4(10) and 117.7(12)
∠ C1–B1–C1b	120.2(1)	118.6(2)	119.0(5) / 117.7(4) / 118.0(4) / 118.1(4)	116.1(5) / 115.9(10) and 118.0(12)
∠ C1a–B1–C1b	121.2(1)	122.2(2)	121.1(5) / 122.7(5) / 122.5(4) / 122.6(4)	125.4(5) / 124.3(5)
Sum ∠ C–B1–C	359.8(3)	359.7(6)	359.5(5) / 359.4(5) / 359.8(5) / 359.6(4)	359.9(5) / 359.6(10) and 360.0(12)
∠ C2a–C1a–B1–C1	−62.4(2)	94.4(2)	93.9(6) / -100.3(6) / 97.4(5) / −95.4(6)	−87.8(7) / 77.0(9)
∠ C2b–C1b–B1–C1	–44.0(2)	−54.9(3)	−56.8(7) / 61.2(6) / −60.1(6) / 58.4(6)	−93.6(6) / 104.6(9)

[a] Compound **CoCp$_2$$^+1^{•-}$** contains 4 symmetry-independent molecules in the unit cell and values for all molecules are given here.

[b] Compound **{Na·(THF)$_6$}$^+$1$^{•-}$** contains 2 symmetry-independent molecules in the unit cell and values for both molecules are given here. One molecule has a disordered tolyl group attached to the B1 atom and values for both parts are given here.

Electrochemistry

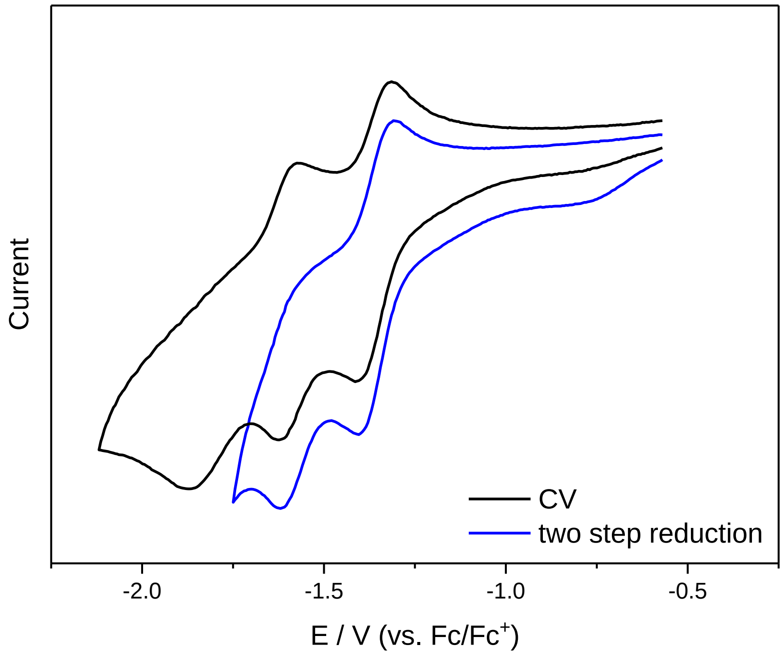

Figure S6: Cyclic voltammograms of the reversible (E_{red} = −1.35 V) and irreversible redox events of **1**. In blue only two reduction events were achieved showing the irreversible nature of the second reduction. The cyclic voltammograms were measured in CH_2Cl_2 with [*n*Bu_4N][PF_6] as the electrolyte with a scan rate of 250 mVs^{-1}. All measurements are referenced to the Fc/Fc^+ ion couple.

Photophysical properties

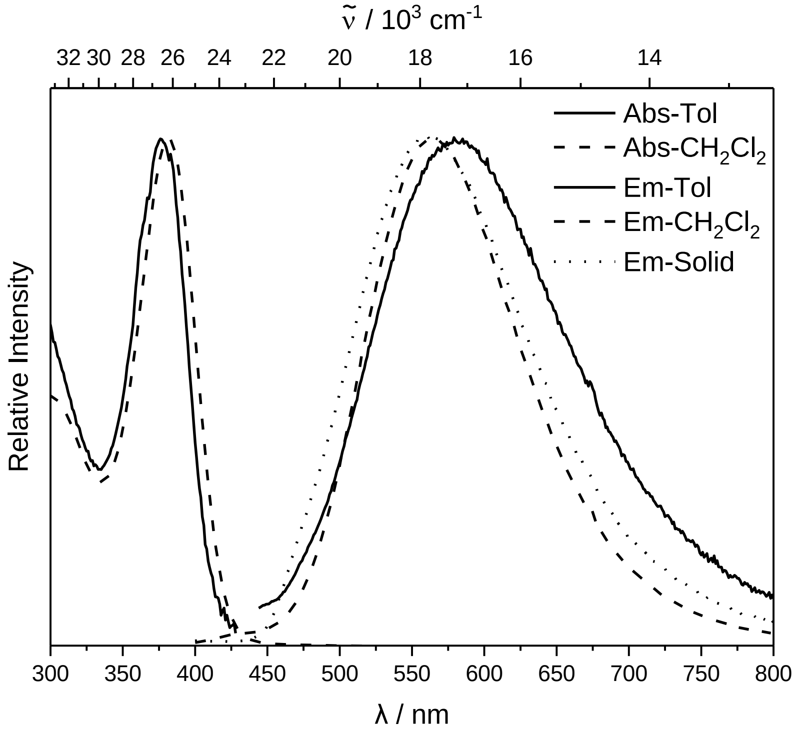

Figure S7: Absorption (<450 nm) and emission (>450 nm, excited at the lowest energy absorption maximum) spectra of **1** in toluene (solid line), CH_2Cl_2 (dashed line) and in the solid state (dotted line).

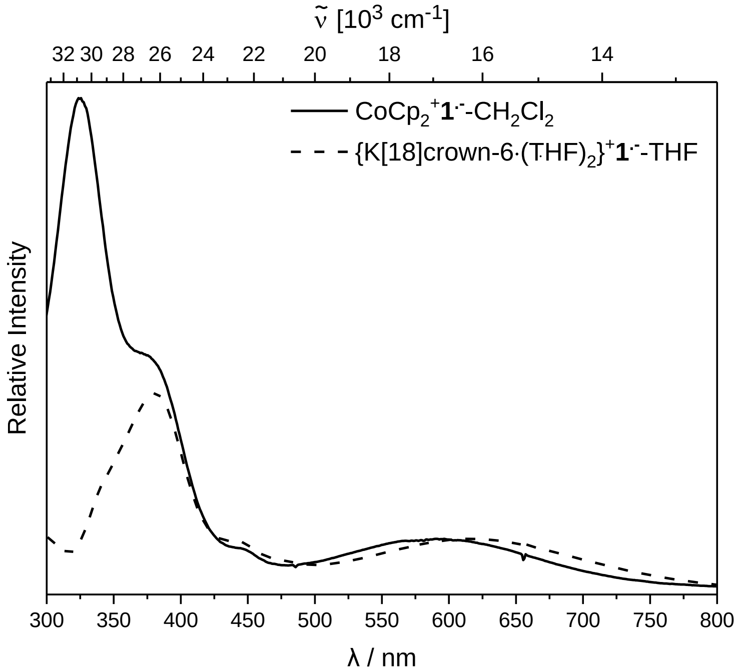

Figure S8: Absorption spectra of $\mathbf{CoCp_2^+1^{\bullet-}}$ (solid line) in CH_2Cl_2 and $\mathbf{\{K[18]crown\text{-}6\cdot(THF)_2\}^+1^{\bullet-}}$ in THF (dashed line).

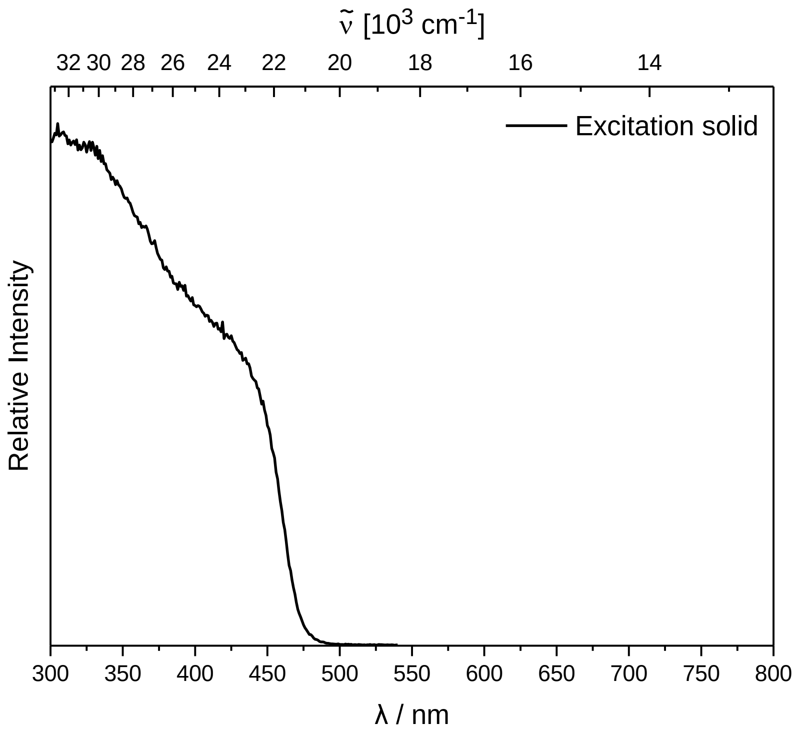

Figure S9: Excitation spectrum of **1** in the solid state.

TD-DFT calculations

1

Table S3: Lowest energy singlet electronic transition of **1** (TD-DFT B3LYP/6-31G*, gas phase).

FC-S_n	E[eV](E[nm])	f	Contribution > 10%	Λ
S_1	3.16 (392)	0.044	HOMO->LUMO (98%)	0.42
S_2	3.50 (354)	0.0036	H–1->LUMO (98%)	0.30
S_3	3.64 (340)	0.0002	H–2->LUMO (99%)	0.28
S_4	3.76 (329)	0.0082	H–4->LUMO (16%)<br>H–3->LUMO (82%)	0.31
S_5	3.89 (319)	0.0067	H–4->LUMO (83%)<br>H–3->LUMO (16%)	0.34

Simulated absorption spectrum at the B3LYP/6-31G* level of theory.

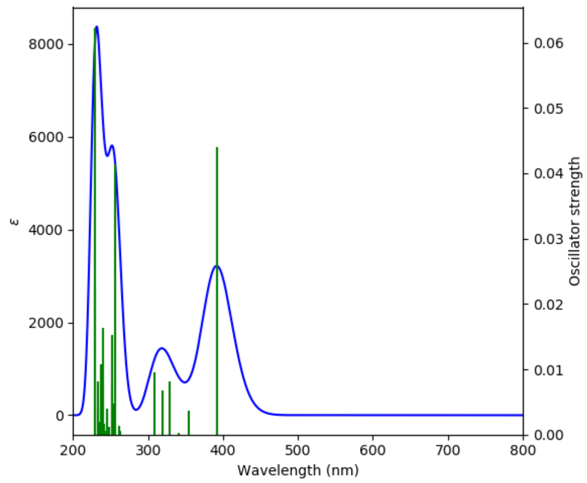

Orbitals relevant to the $S_1 \leftarrow S_0$ transition (B3LYP/6-31G*)

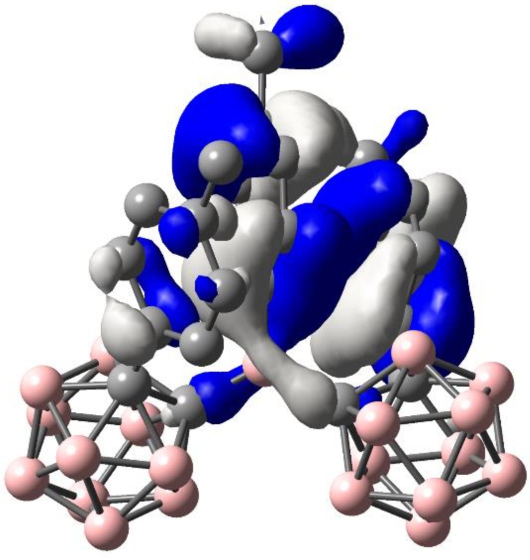

HOMO: −6.64

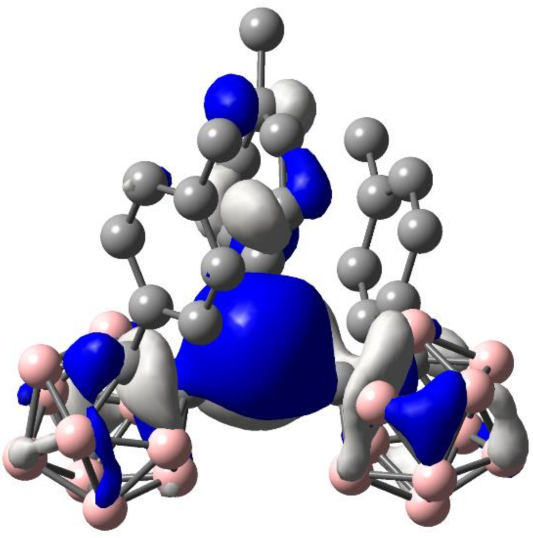

LUMO: −2.75

1

Table S4: Lowest energy singlet electronic transition of **1** (TD-DFT CAM-B3LYP/6-31G*, toluene).

FC-S_n	E[eV] (E[nm])	f	Contribution > 10%	Λ
S_1	3.71 (334)	0.102	HOMO -> LUMO (86%)	0.42
S_2	4.27 (290)	0.004	H–2 -> LUMO (12%)<br>H–1 -> LUMO (76%)	0.33
S_3	4.53 (274)	0.013	H–2 -> LUMO (82%)<br>H–1 -> LUMO (13%)	0.32
S_4	4.61 (269)	0.014	H–4 -> LUMO (11%)<br>H–3 -> LUMO (73%)	0.36
S_5	4.96 (250)	0.006	H–4 -> LUMO (76%)<br>H–3 -> LUMO (10%)	0.30

Table S5: Lowest energy singlet electronic transition of **1** (TD-DFT CAM-B3LYP/6-31G*, CH_2Cl_2).

FC-S_n	E[eV] (E[nm])	F	Contribution > 10%	Λ
S_1	3.64 (340)	0.099	HOMO -> LUMO (86%)	0.41
S_2	4.19 (296)	0.004	H–1 -> LUMO (84)	0.32
S_3	4.48 (277)	0.015	H–2 -> LUMO (88%)	0.30
S_4	4.55 (272)	0.012	H–4 -> LUMO (12%)<br>H–3 -> LUMO (73%)	0.35
S_5	4.89 (253)	0.005	H–4 -> LUMO (76%)<br>H–3 -> LUMO (13%)	0.30

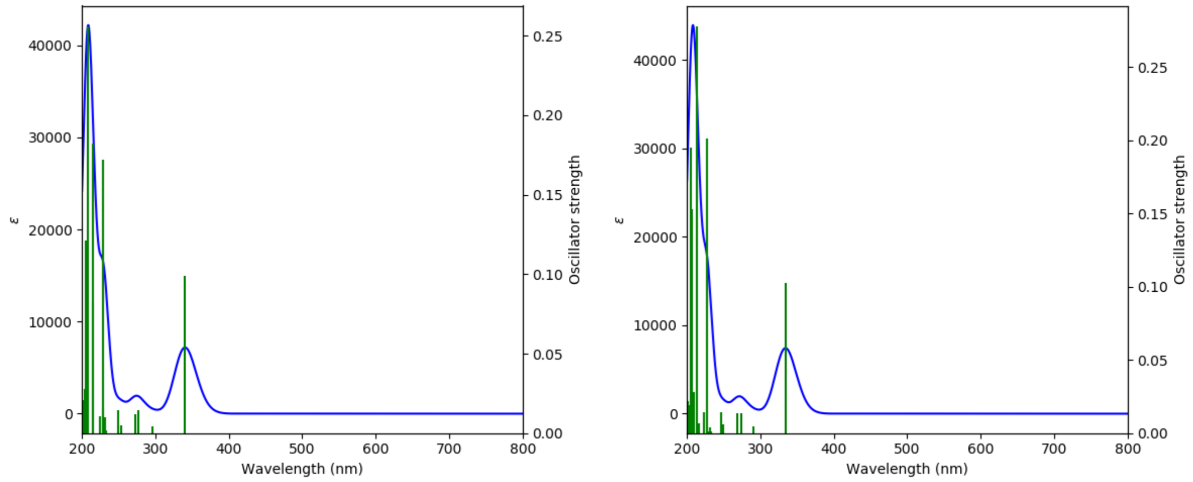

Simulated absorption spectrum at the CAM-B3LYP/6-31G* level of theory (left toluene, right CH_2Cl_2).

Orbitals relevant to the $S_1 \leftarrow S_0$ transition (CAM-B3LYP/6-31G*, toluene)

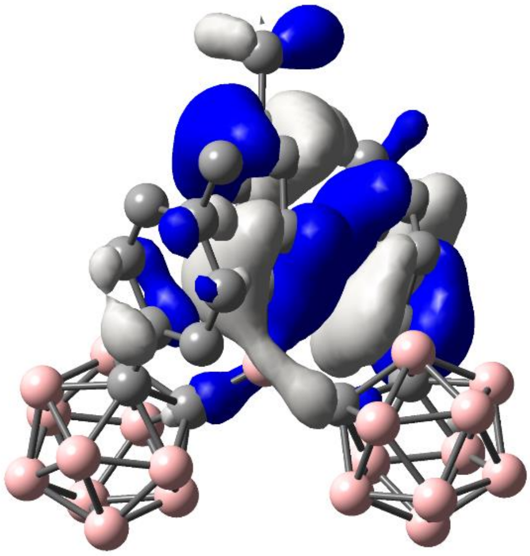

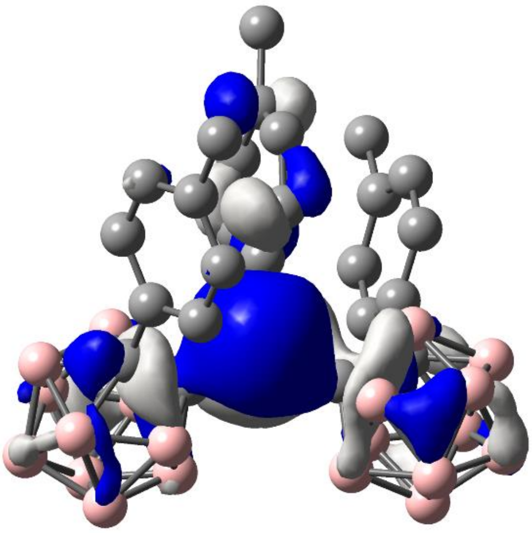

HOMO: −7.92 LUMO: −1.56

Orbitals relevant to the $S_1 \leftarrow S_0$ transition (CAM-B3LYP/6-31G*, CH_2Cl_2)

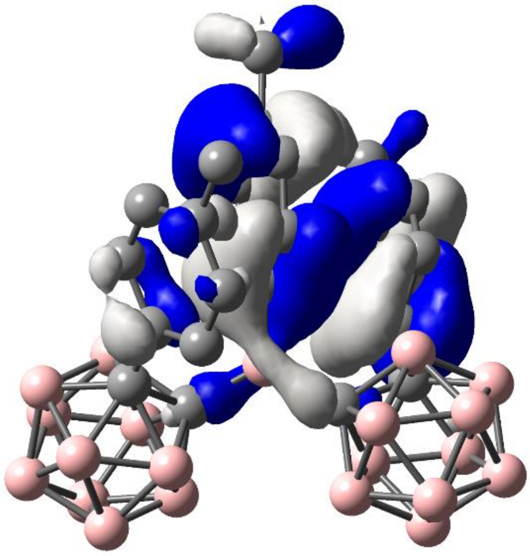

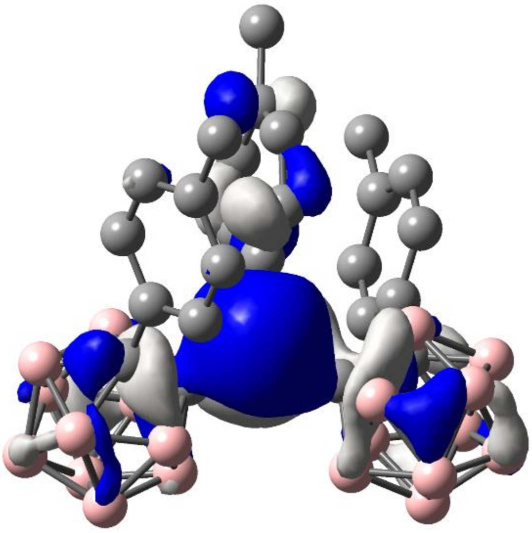

HOMO: −7.84 LUMO: −1.57

$\mathbf{1}^{\bullet-}$

Table S6: Lowest energy singlet electronic transition of $\mathbf{1}^{\bullet-}$ (B3LYP/6-31G*, gas phase).

FC-S_n	E[eV] (E[nm])	f	Contribution > 10%	Λ
S_1	2.08 (596)	0.1301	HOMO α -> LUMO α (92%)	0.68
S_2	2.35 (528)	0.0054	HOMO α -> L+1 α (93%)	0.54
S_3	2.79 (443)	0.0014	HOMO α -> L+2 α (71%)<br>HOMO α -> L+3 α (27%)	0.50
S_4	2.88 (430)	0.003	HOMO α -> L+2 α (27%)<br>HOMO α -> L+3 α (71%)	0.68
S_5	3.13 (396)	0.0042	HOMO α -> L+4 α (35%)<br>HOMO β -> LUMO β (46%)	0.54

Simulated absorption spectrum at B3LYP/6-31G* level of theory.

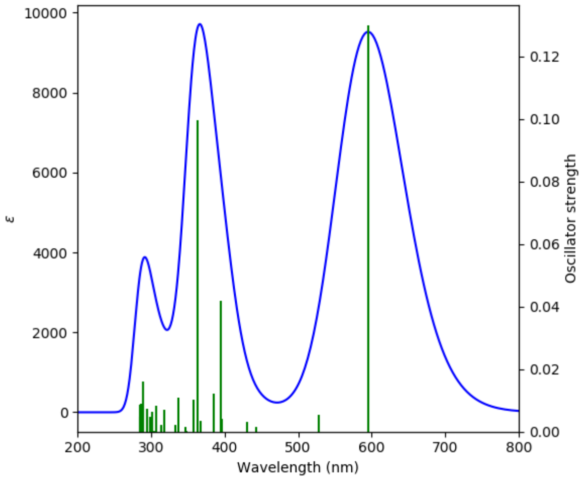

Orbitals relevant to the $S_1 \leftarrow S_0$ transition (B3LYP/6-31G*)

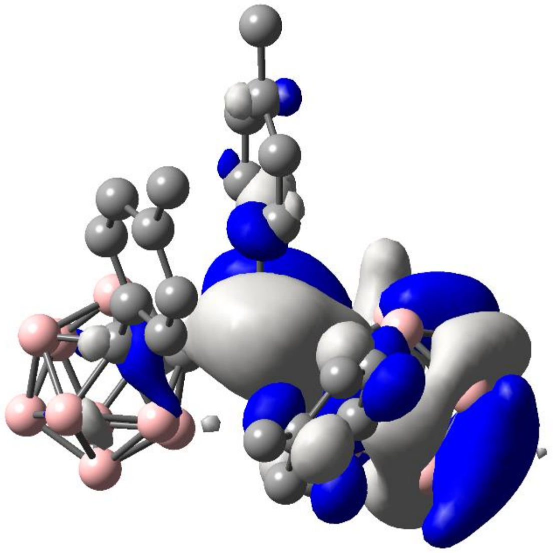

HOMO α: −1.53

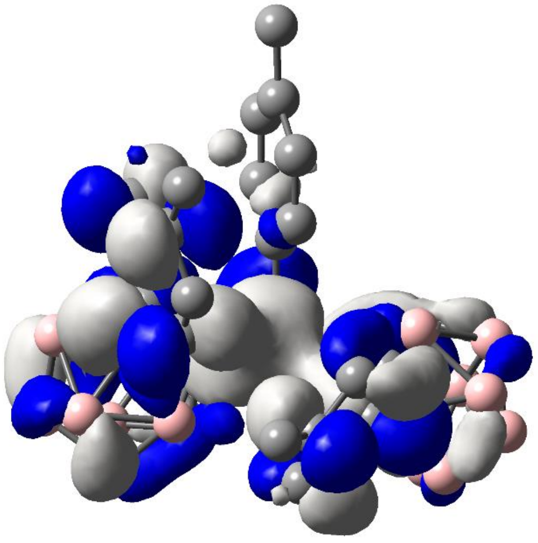

LUMO α: 1.25

Theoretical calculations: Cartesian coordinates

1

DFT B3LYP/6-31G*, gas phase, S_0

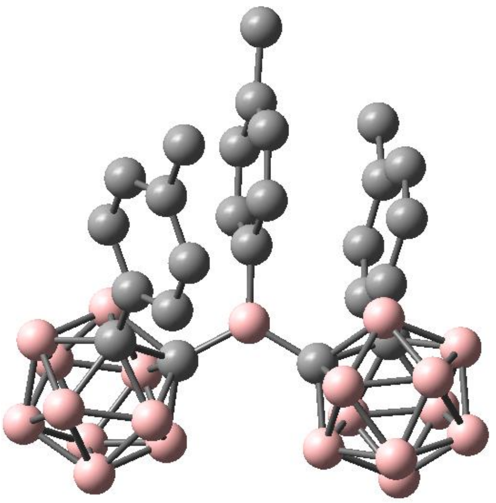

Point group: C1

Total energy: –941,162.143 kcal mol^{-1}

Dipole moment: 9.5768 D

Imaginary frequencies: 0

C 0.29653400 0.94027100 -0.15213800
C 0.84067300 1.50267300 -1.32369400
H 0.89481800 0.91188700 -2.23096000
C 1.34882300 2.79387000 -1.33423700
H 1.78858800 3.18688900 -2.24739000
C 1.29738500 3.59951600 -0.18893400
C 1.88222800 4.98844300 -0.19963300
H 1.49715900 5.59416800 0.62640200
H 1.66237400 5.50928200 -1.13800200
H 2.97528000 4.95170600 -0.09950800
C 0.71121500 3.06632000 0.96271700
H 0.62659100 3.68071900 1.85558500
C 0.23578600 1.75777900 0.98833100
H -0.23028500 1.38899200 1.89457500
C -1.47056600 -0.86605400 -1.12435900
C -2.95027700 -0.10231600 -0.74216800
C -2.98969800 0.79616400 0.46260500
C -2.99532800 0.28438800 1.76467000
H -3.00808900 -0.78386200 1.93142200
C -2.97903000 1.14085200 2.86421400
H -2.96910000 0.71586400 3.86428700
C -2.97692700 2.52976500 2.70215400
C -2.97533900 3.45646300 3.89142000
H -3.99073100 3.80552000 4.11909700
H -2.36251600 4.34465500 3.70395400
H -2.59105400 2.95796300 4.78647700
C -2.99994900 3.03428500 1.39581700
H -2.99783700 4.10934600 1.23657500
C -3.00585700 2.18665300 0.29475100
H -3.00343300 2.60956200 -0.70195800
C 0.50851000 -1.63366500 0.72989000
C 2.26624400 -1.60481700 0.69946300
C 3.00972000 -0.59064200 -0.11612900
C 3.27732400 -0.80051800 -1.47271800
H 2.93215600 -1.70267300 -1.96215600
C 3.99097800 0.14364000 -2.20763100
H 4.18036300 -0.03794000 -3.26223400
C 4.46614200 1.31597500 -1.61320500
C 5.18961300 2.36574500 -2.41679200
H 6.04705700 2.77035500 -1.86823800
H 4.52361700 3.20894400 -2.64336100
H 5.55094400 1.96580400 -3.36883900
C 4.22495800 1.50131900 -0.24611000
H 4.59320900 2.39746800 0.24644900
C 3.51154100 0.56721800 0.49233800
H 3.33360200 0.74424700 1.54514100
B -0.22645900 -0.51973900 -0.16136500
B -2.07209200 0.46360100 -2.09639700
H -1.55051100 1.51007700 -2.01472900
B -3.82398300 0.28724800 -2.15987900
H -4.48090300 1.27089800 -2.12550200
B -4.25815800 -1.11470800 -1.17076600
H -5.20501400 -1.08464400 -0.46138100
B -2.76484300 -1.80516400 -0.51750800
H -2.69327200 -2.22487000 0.57609500
B -1.32126900 -0.96183900 -2.82575100
H -0.23318000 -0.86381000 -3.28657600
B -1.77728100 -2.37626300 -1.87036400
H -1.00437400 -3.24687700 -1.70307500
B -3.53735300 -2.55588600 -1.91386500
H -4.04326000 -3.62268700 -1.80240700
B -4.18540000 -1.25857800 -2.94349300
H -5.17554100 -1.39042600 -3.58407900
B -2.81394800 -0.25982000 -3.50463100
H -2.79622400 0.32171400 -4.53877500
B -2.64164900 -2.02799700 -3.36660400
H -2.50093300 -2.72975900 -4.31293700
B 1.37244300 -2.84896800 -0.10403500
H 1.32404800 -2.85371400 -1.28138400
B -0.00291500 -3.25855200 0.93422800
H -1.00508800 -3.60424000 0.43468000
B 0.01265300 -2.12483500 2.28417000
H -0.97783400 -1.64306200 2.71674700
B 1.43862200 -1.08429200 2.09869700
H 1.43952700 0.05702700 2.36819700
B 1.45230900 -4.24777500 0.98676500
H 1.45264200 -5.34630800 0.53995300
B 0.60792500 -3.79267900 2.49435800
H -0.01094700 -4.57389200 3.13818000
B 1.49209000 -2.43024200 3.22636800
H 1.51923600 -2.22010500 4.39391100
B 2.89175300 -2.08879900 2.20561400
H 3.89934500 -1.56924900 2.54419800
B 2.85979200 -3.18877800 0.81656300
H 3.84502300 -3.42609400 0.20573700
B 2.37694400 -3.76636100 2.42987000
H 3.06044100 -4.52670000 3.03221200

1(S$_1$)

DFT B3LYP/6-31G*, gas phase, S$_1$

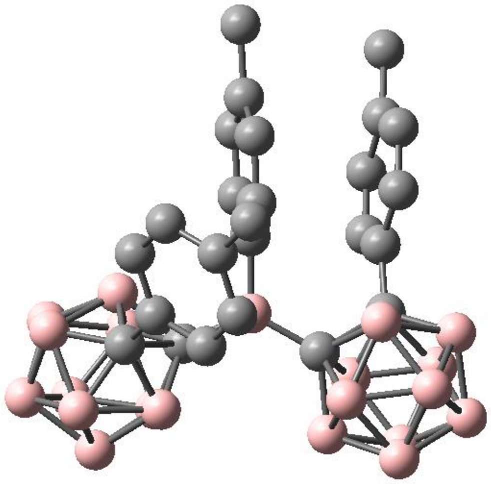

Point group: C1

Total energy: –940,356.149 kcal mol^{-1}

Dipole moment: 17.7779 D

Imaginary frequencies: 0

C	0.45644255	0.83132640	-0.47167087
C	1.20514537	1.37313004	-1.56384542
H	1.35675199	0.75599538	-2.43843505
C	1.69911730	2.64595285	-1.52641958
H	2.26144484	3.04268464	-2.36501346
C	1.48169848	3.46782512	-0.39644601
C	2.02217494	4.85697037	-0.37195224
H	2.06855936	5.25984816	0.64151902
H	1.37257563	5.51587572	-0.96307672
H	3.01645233	4.90260497	-0.82555405
C	0.71199861	2.97343764	0.67858885
H	0.52134681	3.61376900	1.53310793
C	0.18961207	1.71002781	0.63453647
H	-0.42542680	1.34887444	1.44891329
C	-1.42972112	-0.69135959	-1.36645761
C	-3.25228352	0.15586011	-0.54459854
C	-3.15696073	0.77686215	0.79829808
C	-3.00219470	0.01243350	1.95504566
H	-3.02543086	-1.06608610	1.88903523
C	-2.81163018	0.61687188	3.19212915
H	-2.67879719	-0.00809097	4.07083293
C	-2.79068850	2.00344142	3.32409149
C	-2.61628929	2.65483474	4.66978183
H	-3.58487973	2.81150700	5.15813739
H	-2.13570219	3.63366771	4.58280961
H	-2.01178212	2.03555857	5.33822049
C	-2.97370481	2.76844488	2.16944448
H	-2.97654935	3.85296512	2.24251997
C	-3.14921268	2.17078030	0.93222785
H	-3.27251635	2.79000465	0.05166733
C	0.52123885	-1.80302605	0.29856303
C	2.19984536	-1.72734932	0.51078000
C	3.01555384	-0.58077647	-0.01957547
C	3.56878685	-0.62035677	-1.30731183
H	3.34653391	-1.45419011	-1.96047968
C	4.40860779	0.38759632	-1.74946906
H	4.82188112	0.33269462	-2.75228456
C	4.73449053	1.46835107	-0.92819156
C	5.66297451	2.55016488	-1.40486033
H	5.50866256	3.48236129	-0.85421546
H	5.52937302	2.75206086	-2.47134633
H	6.70869924	2.25661058	-1.25983449
C	4.20007363	1.49415282	0.36184903
H	4.45415874	2.31280246	1.02909603
C	3.36869184	0.48414767	0.81483865
H	2.99299016	0.51908928	1.82877260
B	-0.16084262	-0.61473728	-0.52924476
B	-2.22194083	0.82296122	-1.72724843
H	-1.64730031	1.82991596	-1.46848519
B	-4.02604547	0.84410492	-1.80855045
H	-4.62166161	1.84911310	-1.60397689
B	-4.47842106	-0.74376524	-1.15803274
H	-5.41080964	-0.93801479	-0.45168470
B	-2.87855174	-1.48913289	-0.76316220
H	-2.76902912	-2.20154569	0.17106060
B	-1.59320653	-0.29731411	-2.96208727
H	-0.59127160	-0.06264201	-3.55553828
B	-2.03411625	-1.89041317	-2.31078294
H	-1.37784687	-2.86611262	-2.43313442
B	-3.79300523	-1.96913851	-2.22282240
H	-4.32777149	-3.02353176	-2.32879021
B	-4.46675881	-0.46518908	-2.91448281
H	-5.48630593	-0.42530249	-3.52224979
B	-3.07879511	0.58951477	-3.27684950
H	-3.09237786	1.40967740	-4.13622378
B	-3.00752392	-1.15581945	-3.59782762
H	-2.97663813	-1.61462018	-4.69317448
B	1.53694110	-2.86836045	-0.57827385
H	1.64179954	-2.68486923	-1.73952711
B	0.05429303	-3.43976296	0.17695884
H	-0.86024039	-3.70594799	-0.51187422
B	-0.16294551	-2.55358580	1.67652569
H	-1.22387411	-2.18355769	2.03132841
B	1.18527723	-1.44788806	1.84921490
H	1.07125038	-0.34740090	2.25888871
B	1.53704350	-4.39309699	0.29855679
H	1.65962886	-5.41248586	-0.29443569
B	0.47774733	-4.19797474	1.71180981
H	-0.17588658	-5.08997805	2.14042713
B	1.17894133	-2.94795076	2.75807500
H	1.04111649	-2.91937876	3.93618504
B	2.66751950	-2.39631488	2.00212011
H	3.59769157	-1.89628619	2.53724433
B	2.88894528	-3.28189448	0.48908693
H	3.96733114	-3.37430836	0.00877841
B	2.23140060	-4.10266855	1.90912485
H	2.87146964	-4.91966124	2.48399420

1$^{\bullet-}$

DFT B3LYP/6-31G*, gas phase, S_0

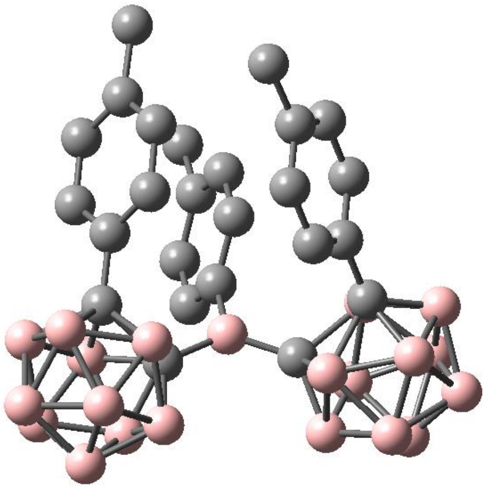

Point group: C1

Total energy: –941211.546 kcal mol^{-1}

Dipole moment: 14.4146 D

Imaginary frequencies: 0

C 0.01904300 -2.13318700 -0.28532200
C 1.47975800 -1.78244200 -1.17838200
B -0.04926100 -1.72783900 -1.95140300
H -0.50413500 -0.68814100 -2.25921200
B 1.50319000 -2.97454000 0.04327200
H 2.07644600 -2.74776400 1.04348100
B -0.04463500 -3.82043600 -0.02827700
H -0.53311600 -4.19425100 0.98488800
B -1.00612200 -3.06089300 -1.29411500
H -2.16807700 -2.93234700 -1.18603500
B 1.38194300 -2.42444600 -2.74949700
H 1.90092700 -1.79871200 -3.61254800
B 2.35042100 -3.21011200 -1.49509600
H 3.53260600 -3.12607100 -1.50491600
B 1.40253900 -4.52190100 -0.79014800
H 1.93405400 -5.48152600 -0.33104300
B -0.17410000 -3.25447800 -2.84115100
H -0.78444400 -3.30376100 -3.85986300
B 1.31403800 -4.18739400 -2.54247700
H 1.79038300 -4.91350900 -3.35577500
B -0.16026600 -4.57287000 -1.63805100
H -0.76986000 -5.58140200 -1.79573800
C 2.22757400 -0.50643700 -0.92451800
C 1.82073800 0.71014800 -1.48008300
H 0.92954500 0.76671700 -2.09130200
C 2.54349700 1.87532700 -1.23048400
H 2.18912600 2.81086900 -1.64994600
C 3.68477600 1.86502200 -0.42659500
C 4.10218000 0.63879700 0.10376000
H 4.98672000 0.59878400 0.73500500
C 3.38835600 -0.52641300 -0.13971500
H 3.72210100 -1.45728700 0.30050700
C 4.42043500 3.13967800 -0.09814800
H 5.50721700 3.00232100 -0.14155100
H 4.17776400 3.47766900 0.91821500
H 4.15047300 3.94655500 -0.78719000
B -0.48957400 -1.06199600 0.81502100
C 0.56625700 -0.46292700 1.82098000
C 0.99346600 0.87420900 1.74532800
H 0.60445300 1.51382700 0.96232000
C 1.93170700 1.39456200 2.63021300
H 2.24687100 2.43148300 2.52630200
C 2.48174400 0.60546400 3.64663200
C 2.04060900 -0.71652700 3.75584000
H 2.43393800 -1.34796200 4.55050500
C 1.10506300 -1.23738100 2.86138900
H 0.78377900 -2.26871400 2.98122500
C 3.53588900 1.16001100 4.57380400
H 4.52726700 1.16143800 4.09891100
H 3.61514500 0.56665000 5.49125100
H 3.31575000 2.19557300 4.85925500
C -1.95741100 -0.66138200 0.95302800
C -2.81528200 1.22284400 -0.01199500
B -2.33517300 0.95321300 1.60466100
H -1.45155900 1.59649200 2.05139500
B -3.06118300 -0.42339300 -0.40085200
H -2.72901300 -0.79497500 -1.47000700
B -3.86776500 1.81374100 1.09641000
H -3.95883400 2.99772000 1.17282600
B -4.37948200 0.84346000 -0.31363400
H -4.86208800 1.29034200 -1.30501300
B -3.30691800 -1.59386500 0.96806200
H -3.15166200 -2.76436500 0.85701600
B -2.78253500 -0.61361500 2.37567900
H -2.20923900 -1.02788500 3.33109500
B -3.88525300 0.76178600 2.50792200
H -4.08205700 1.23184500 3.58481500
B -4.70718900 -0.79490800 0.25048900
H -5.51074800 -1.45974300 -0.32471800
B -4.51728800 -0.83695500 2.02927100
H -5.18628300 -1.51841800 2.74227600
B -5.16454500 0.60811600 1.26504500
H -6.29196900 0.95608800 1.43423000
C -1.96626500 2.05080000 -0.88892200
C -1.88780000 1.80535700 -2.27118200
H -2.47055100 0.99706300 -2.69672100
C -1.07973900 2.58212200 -3.09465800
H -1.04025500 2.36435800 -4.16002100
C -0.31661200 3.63848000 -2.57892500
C -0.40412600 3.89203100 -1.20555700
H 0.17045900 4.71040100 -0.77632400
C -1.20762600 3.11501500 -0.37497800
H -1.25839800 3.33463000 0.68530000
C 0.58849200 4.45084300 -3.47248200
H 0.11103200 4.67203100 -4.43415600
H 1.52139900 3.91405900 -3.69449200
H 0.86229000 5.40244100 -3.00407000

References

[1] A. Toppino, A. R. Genady, M. E. El-Zaria, J. Reeve, F. Mostofian, J. Kent, J. F. Valliant, *Inorg. Chem.* **2013**, *52*, 8743-8749.

[2] G. R. Fulmer, A. J. M. Miller, N. H. Sherden, H. E. Gottlieb, A. Nudelman, B. M. Stoltz, J. E. Bercaw, K. I. Goldberg, *Organometallics* **2010**, *29*, 2176-2179.

[3] G. M. Sheldrick, *Acta Crystallogr. Sect. A* **2015**, *71*, 3-8.

[4] G. M. Sheldrick, *Acta Crystallogr. Sect. A* **2008**, *64*, 112-122.

[5] C. B. Hübschle, G. M. Sheldrick, B. Dittrich, *J. Appl. Cryst.* **2011**, *44*, 1281-1284.

[6] A. Spek, *Acta Crystallogr. C* **2015**, *71*, 9-18.

[7] K. Brandenburg, H. Putz, Diamond Version 4.2.0. Crystal and Molecular Structure Visualization, Crystal Impact, Bonn (Germany), **2016**.

[8] Gaussian 09, Revision 9.E.01, M. J. Frisch, G. W. Trucks, H. B. Schlegel, G. E. Scuseria, M. A. Robb, J. R. Cheeseman, G. Scalmani, V. Barone, G. A. Petersson, H. Nakatsuji, X. Li, M. Caricato, A. V. Marenich, J. Bloino, B. G. Janesko, R. Gomperts, B. Mennucci, H. P. Hratchian, J. V. Ortiz, A. F. Izmaylov, J. L. Sonnenberg, D. Williams-Young, F. Ding, F. Lipparini, F. Egidi, J. Goings, B. Peng, A. Petrone, T. Henderson, D. Ranasinghe, V. G. Zakrzewski, J. Gao, N. Rega, G. Zheng, W. Liang, M. Hada, M. Ehara, K. Toyota, R. Fukuda, J. Hasegawa, M. Ishida, T. Nakajima, Y. Honda, O. Kitao, H. Nakai, T. Vreven, K. Throssell, J. A. Montgomery, Jr., J. E. Peralta, F. Ogliaro, M. J. Bearpark, J. J. Heyd, E. N. Brothers, K. N. Kudin, V. N. Staroverov, T. A. Keith, R. Kobayashi, J. Normand, K. Raghavachari, A. P. Rendell, J. C. Burant, S. S. Iyengar, J. Tomasi, M. Cossi, J. M. Millam, M. Klene, C. Adamo, R. Cammi, J. W. Ochterski, R. L. Martin, K. Morokuma, O. Farkas, J. B. Foresman, and D. J. Fox, Gaussian, Inc., Wallingford CT, **2016**.

[9] T. Lu, F. Chen, *J. Comput. Chem.* **2012**, *33*, 580-592.

[10] Lee, Yang, Parr, *Phys. Rev. B* **1988**, *37*, 785-789.

[11] G. A. Petersson, M. A. Al-Laham, *J. Chem. Phys.* **1991**, *94*, 6081-6090.

[12] G. A. Petersson, A. Bennett, T. G. Tensfeldt, M. A. Al-Laham, W. A. Shirley, J. Mantzaris, *J. Chem. Phys.* **1988**, *89*, 2193-2218.

[13] S. Grimme, *Chem. Eur. J.* **2004**, *10*, 3423-3429.

[14] T. Yanai, D. P. Tew, N. C. Handy, *Chem. Phys. Lett.* **2004**, *393*, 51-57.

[15] M. J. G. Peach, P. Benfield, T. Helgaker, D. J. Tozer, *J. Chem. Phys.* **2008**, *128*, 044118.
